# Influence of In Vitro IL-2 or IL-15 Alone or in Combination with Hsp 70 Derived 14-Mer Peptide (TKD) on the Expression of NK Cell Activatory and Inhibitory Receptors on Peripheral Blood T Cells, B Cells and NKT Cells

**DOI:** 10.1371/journal.pone.0151535

**Published:** 2016-03-16

**Authors:** Ilona Hromadnikova, Shuang Li, Katerina Kotlabova, Anne M. Dickinson

**Affiliations:** 1 Department of Molecular Biology and Cell Pathology, Third Faculty of Medicine, Charles University, Prague, Ruska 87, 10 000 Prague 10, Czech Republic; 2 Haematological Sciences, Institute of Cellular Medicine, Newcastle University, Newcastle upon Tyne, NE2 4HH, United Kingdom; Nottingham Trent University, UNITED KINGDOM

## Abstract

Previous studies from Multhoff and colleagues reported that plasma membrane Hsp70 acts as a tumour-specific recognition structure for activated NK cells, and that the incubation of NK cells with Hsp70 and/or a 14-mer peptide derived from the N-terminal sequence of Hsp70 (TKDNNLLGRFELSG, TKD, aa 450–463) plus a low dose of IL-2 triggers NK cell proliferation and migration, and their capacity to kill cancer cells expressing membrane Hsp70. Herein, we have used flow cytometry to determine the influence of in vitro stimulation of peripheral blood mononuclear cells from healthy individuals with IL-2 or IL-15, either alone or in combination with TKD peptide on the cell surface expression of CD94, NK cell activatory receptors (CD16, NK2D, NKG2C, NKp30, NKp44, NKp46, NKp80, KIR2DL4, DNAM-1 and LAMP1) and NK cell inhibitory receptors (NKG2A, KIR2DL2/L3, LIR1/ILT-2 and NKR-P1A) by CD3+CD56+ (NKT), CD3+CD4+, CD3+CD8+ and CD19+ populations. NKG2D, DNAM-1, LAMP1 and NKR-P1A expression was upregulated after the stimulation with IL-2 or IL-15 alone or in combination with TKD in NKT, CD8+ T cells and B cells. CD94 was upregulated in NKT and CD8+ T cells. Concurrently, an increase in a number of CD8+ T cells expressing LIR1/ILT-2 and CD4+ T cells positive for NKR-P1A was observed. The proportion of CD8+ T cells that expressed NKG2D was higher after IL-2/TKD treatment, when compared with IL-2 treatment alone. In comparison with IL-15 alone, IL-15/TKD treatment increased the proportion of NKT cells that were positive for CD94, LAMP1 and NKRP-1A. The more potent effect of IL-15/TKD on cell surface expression of NKG2D, LIR1/ILT-2 and NKRP-1A was observed in B cells compared with IL-15 alone. However, this increase was not of statistical significance. IL-2/TKD induced significant upregulation of LAMP1 in CD8+ T cells compared with IL-2 alone. Besides NK cells, other immunocompetent cells present within the fraction of peripheral blood mononuclear cells were influenced by the treatment with low-dose interleukins themselves or in combination with hsp70 derived (TKD) peptide.

## 1. Introduction

Previous studies from Multhoff and colleagues have reported the selective expression of membrane form of the 70 kDa heat shock protein Hsp70 on tumour cells, including leukaemia blasts, but not on corresponding non-malignant tissues or non-transformed cells [1, 2, 3]. Moreover, a membrane Hsp70 positive tumor phenotype has been found to be associated with a significantly decreased overall survival in patients with lower rectal and lung carcinomas [4]. Plasma membrane Hsp70 was demonstrated to act as a tumor-specific recognition structure for pre-activated NK cells expressing high amounts of CD94. Furthermore, Hsp70 plasma membrane expression correlates with an increased sensitivity to allogeneic NK cells [2, 5]. The epitope recognized by cmHsp70.1 monoclonal antibody, 8-mer peptide NLLGRFEL (NLL, comprising amino acids 454–461), was found to be localized within C-terminal substrate binding domain of the inducible Hsp70 molecule exposed to the extracellular milieu of tumors [6, 7]. Multhoff et al. [8] demonstrated that similar to full-length Hsp70 protein, the N-terminal-extended 14-mer peptide TKDNNLLGRFELSG (TKD, aa 450–463) was able to stimulate the cytolytic and proliferative activity of NK cells at concentrations equivalent to full-length Hsp70 protein. Gastpar et al. [9] previously shown that the incubation of lymphocytes with Hsp70-derived peptide TKD in the presence of a low dose of IL-2 results in an enhanced cytolytic and migratory capacity of NK cells toward Hsp70 membrane-positive tumor cells in vitro and in a xenograft tumor mouse model [10]. In a clinical phase I trial, the tolerability, feasibility, and safety of adoptively transferred, autologous IL-2/TKD-activated NK cells have been shown in patients having colorectal and lung carcinoma [4, 11].

We have recently reported the effect of in vitro stimulation with IL-2 or IL-15 alone or in combination with Hsp70 derived 14-mer peptide (TKD) on cell surface expression of NK activatory and inhibitory receptors in CD3^-^CD56^+^ cellular population within peripheral blood mononuclear cells of healthy individuals [12]. The cell surface expression profile of the following activatory receptors was studied using flow cytometry: a low affinity receptor for aggregated IgG (CD16), members of NKG2 natural killer cell receptor family (NKG2D/CD314 and NKG2C associating with CD94 to form a heterodimer), members of the natural cytotoxicity receptor (NCR) family (NCR1/NKp46, NCR2/NKp44 and NCR3/NKp30), a killer cell immunoglobulin-like receptor (KIR2DL4/CD158d), a killer cell lectin-like receptor subfamily F, member 1 (KLRF1/NKp80), DNAX accessory molecule-1 (DNAM-1/CD226) and lysosome-associated membrane protein-1 (LAMP1/CD107a) [12]. Additionally, the cell surface expression of the following NK cell inhibitory receptors was determined: NKG2A creating a complex with CD94 molecule, a killer cell immunoglobulin-like receptor (KIR2DL2/L3/CD158b, NKAT2), a member of the leukocyte immunoglobulin-like receptor (LIR) family such as the immunoglobulin-like transcript 2 (LIR1/ILT-2/CD85j) and a killer cell lectin-like receptor subfamily B, member 1 (KLRB1) also known as NKR-P1A/CD161 [12].

Both, NK activatory (CD94, NKG2D, NKp44, NKp30, KIR2DL4, DNAM-1, LAMP1) and NK inhibitory receptors (NKG2A and NKR-P1A) were upregulated after the stimulation with IL-2 or IL-15 alone or in combination with TKD in CD3^-^CD56^+^ cellular population within peripheral blood mononuclear cells of healthy individuals. KIR2DL2/L3, NK inhibitory receptor, was upregulated only by IL-15 and IL-15/TKD. Concurrently, an increase in a number of NK cells positive for NK activatory receptors (CD94, NKp44, NKp30, KIR2DL4, and LAMP1) was observed. IL-15 and IL-15/TKD caused also cell number rise positive for NK inhibitory receptors (KIR2DL2/L3 and NKR-P1A). Cell number positive for NKG2C and NKG2A was increased only by IL-2 and IL-2/TKD. The diverse effect of IL-2 or IL-15 w or w/o TKD on cell surface expression was observed in CD16, NKp46, and LIR1/ILT-2. Although NK cell cytotoxicity finally depends on the type and the expression of ligands on the target cells interacting with NK cell activatory and inhibitory receptors, the study showed clearly how NK cells were influenced under in vitro conditions in the presence of other immunocompetent cells by low-dose interleukins themselves or in combination with TKD peptide [12].

The natural killer receptors are also expressed on other cell types found in human peripheral blood including CD4^+^ and CD8^+^ T cells, B cells and monocytes. Recent data of Strauss-Albee et al. indicated that the expression of NK receptors is coordinately regulated on divergent cell types as the human immune system maturates [13]. NK receptors are more likely to be found on non-NK cells, especially CD8^+^ T cells. While maturing CD8^+^ T cells acquire a specific subset of both, NK inhibitory and activatory receptors (KIRs, CD94/NKG2A, LILRB2A, 2B4 and NKp30), monocytes and B cells show progressively less LILRB1 expression in more mature repertoires [13]. Maturity-driven NK receptor expression on non-NK cell type is influenced by multiple factors including viral infections and particular microenviromental conditions [13, 14]. These receptors may exert an inhibitory activity on T cell receptor-mediated functions and provide an important mechanism of down-regulation of T cell responses, which may be helpful for the maintenance of homeostasis, prevention of autoimmunity, but could be harmful to the host in certain chronic viral infections [14]. Moreover, the finding of NK inhibitory positive receptor immunoregulatory T cells at the tumor site and in the circulation of cancer patients may explain the dysfunction of T lymphocytes against cancer cells, which can cause escape of tumors from immunosurveillance. Significantly increased number of CD4^+^NKR-P1A^+^ T cells was detected in patients with diverse types of cancer and positively correlated with disease stage [15].

On the other side, lowered threshold for monocyte and B cell activation by decreasing inhibitory capacity through decreased LILRB1 expression helps promote inflammatory responses as CD8+ T cells become more difficult to activate [13].

Cytokines are required to induce expression of NK receptors. It has been reported that in vitro stimulation with IL-2, IL-12 and IL-15 cytokines induced the expression of certain NK cell receptors (NKG2D, CD94/NKG2A) on T cells [16, 17]. It is also possible that the cytokines required for NK receptor expression may be produced, at least in part, by T cells themselves at given stages of activation [14].

In the present study we determined the effect of in vitro stimulation using IL-2 and/or IL-15 alone or in combination with stress-inducible Hsp70 derived 14-mer peptide (TKD) on cell surface expression of NK cell activatory and inhibitory receptors in other cell populations within peripheral blood mononuclear cells of healthy individuals. The cell surface expression profile of the activatory and inhibitory receptors was studied in unstimulated and stimulated CD4^+^ T cells, CD8^+^ T cells, CD19^+^ B cells and CD3^+^ CD56^+^ NKT cells.

## 2. Materials and Methods

The study cohort consisted of 52 Caucasian healthy individuals (26 males and 26 females, age range 23–40 years, age mean 32.1 years, age median 31.5 years). Nine millilitres of peripheral blood were collected into EDTA tubes. Peripheral blood mononuclear cells (PBMC) were isolated by density gradient centrifugation using Ficoll-Paque (Amersham Biosciences, Little Chalfont, UK). All healthy individuals who participated in this study provided written informed consent. The study was approved by the Ethics Committee of the Third Faculty of Medicine, Charles University in Prague.

PBMC at concentration of 5x10^6^ were cultured in 5ml RPMI 1640 medium (Cambrex Bio Sciences Verviers, Verviers, Belgium) supplemented with heat-inactivated 5% (v/v) fetal calf serum (FCS, Sigma Biosciences, St. Louis, MO, USA), 6 mM L-glutamine (Gibco Invitrogen Corporation, Carlsbad, CA, USA) and antibiotics (100 U/ml penicillin and 100 μg/ml streptomycin, Sigma Biosciences, St. Louis, MO, USA) at 37°C in a humidified atmosphere of 5% CO_2_ in the presence or absence of recombinant human cytokines IL-2 (100 IU/ml, Sigma Biosciences, St. Louis, MO, USA) or IL-15 (10 IU/ml, Sigma Biosciences, St. Louis, MO, USA) and TKD peptide (2 μg/ml, Apigenex, Czech Republic) from 1 to 5 days. The 14-mer TKD peptide (TKDNNLLGRFELSG; aa 450–463; lot no: LD13-6 hsp70; MW: 1562.8; purity >90%; tare: 27.8118 g; weight: 100 mg) was provided on the basis of the sequence which was originally published by Multhoff et al. [8] and patented by Multimmune GmbH, Munich, Germany.

### 2.1 Flow cytometry analysis of cell surface expression of NK receptors

Flow cytometry was performed on days +1, +3 and +5 after stimulation with interleukin and TKD peptide (day 0) using a standard direct immunofluorescence technique and mouse anti-human monoclonal antibodies conjugated with fluorescein isothiocyanate (FITC), phycoerythrin (PE) and allophycocyanin (APC) on a FACSCalibur^™^ (Becton Dickinson, San Jose, USA). The experiment was settled in such a way that unstimulated cells, cells stimulated with interleukin itself (IL-2 or IL-15) and cells stimulated with the combination of interleukin and TKD peptide (IL-2 + TKD or IL-15 + TKD) were analysed at the same time on each day.

After washing in PBS containing 10% FCS (Sigma Biosciences, St. Louis, MO, USA), single-cell suspension of 0.5 X 10^6^ cells per tube was stained with FITC or APC-conjugated monoclonal antibodies against CD3 (BD Pharmingen, clone: UCHT1 and Exbio Prague, Czech Republic, clone: MEM-57), FITC or PE-conjugated monoclonal antibodies against CD4 (BD Pharmingen, clone: RPA-T4 and BD Biosciences, clone: SK3), FITC or PE-conjugated monoclonal antibodies against CD8 (BD Pharmingen, clone: RPA-T8 and BD Biosciences, clone: SK1), FITC or PE-conjugated monoclonal antibodies against CD56 (Exbio, clone: MEM-188 and BD Pharmingen, clone: B159), FITC-conjugated monoclonal antibodies against appropriate NK cell activatory or inhibitory receptor (NKR-P1A: BD Pharmingen, clone DX12; DNAM-1: RD Systems, clone 102511), PE-conjugated monoclonal antibodies against appropriate NK cell activatory or inhibitory receptor (NKG2D: BD Pharmingen, clone 1D11; LAMP-1: RD Systems, clone 508921; NKG2A: RD Systems, clone 131411; NKG2C: RD Systems, clone 134591; NKp30: RD Systems, clone 210845; NKp44: RD Systems, clone 253415; NKp46: BD Pharmingen, clone 9E2/Nkp46; CD16: Exbio, clone LNK16; CD94: BioLegend, clone DX22; KIR2DL2L3: BioLegend, clone DX27; KIR2DL4: RD Systems, clone 181703) or APC-conjugated monoclonal antibodies against appropriate NK cell activatory or inhibitory receptor (ILT-2: RD Systems, clone 292305; NKp80: RD Systems, clone 239127) for 30 min on ice. In case of B cells PE or APC-conjugated monoclonal antibodies against CD19 (BD Pharmingen, clones: HIB19) were used.

Mouse IgG1-FITC BD Biosciences (BD Biosciences, clone: X40), IgG1-PE (BD Biosciences, clone X40), IgG1-APC (BD Pharmingen, clone MOPC-21), IgG2a-FITC (Exbio Prague, Czech Republic, clone PPV-04) and IgG2a-APC (RD Systems, clone 20102), IgG2b-PE (RD Systems, clone 508921) and IgG2a-PE (RD Systems, clone 131411) were used as isotype-matched controls.

The percentage of positive stained cells was determined as the number of positively stained cells minus the number of cells stained with an isotype-matched negative control antibody. The median fluorescence intensity (MFI) was determined to demonstrate changes in cell surface expression of NK activatory and inhibitory receptors. Only 7-amino-actinomycin D (7-AAD, Becton Dickinson, San Jose, USA) negative, viable cells with intact cell membranes were gated and analysed.

High viability of unstimulated lymphocytes (d1: range 97.0%-99.9%, mean 98.6%; d5: range 97.4%-99.8%, mean 98.6%) and stimulated lymphocytes over the culture period was detected (d5 IL-2 stimulated cells: range 97.2%-99.6%, mean 98.6%; d5 IL-2+TKD stimulated cells: range 97.3%-99.7%, mean 98.7%; d5 IL-15 stimulated cells: range 97.2%-99.8%, mean 98.7%; d5 IL-15+TKD stimulated cells: range 97.2%-99.8%, mean 98.7%).

Similarly, high overall viability of the unstimulated population (d1: range 95.6%-99.1%, mean 97.7%; d5: range 95.0%-98.1%, mean 96.3%) and stimulated population over the culture period was detected (d5 IL-2 stimulated cells: range 96.0%-99.1%, mean 97.7%; d5 IL-2+TKD stimulated cells: range 96.1%-99.1%, mean 97.7%; d5 IL-15 stimulated cells: range 95.6%-99.1%, mean 97.6%; d5 IL-15+TKD stimulated cells: range 95.9%-99.1%, mean 97.7%).

### 2.2 Statistical analysis

Normality of the data was assessed using Shapiro-Wilk test, which indicated that our data did follow a normal distribution. Therefore, proportions of cells expressing an appropriate antigen and the amount of appropriate receptor expressed by cells of interest were compared between groups by parametric test (Student´s t-test) using SPSS Statistics software (version 21.0; IBM, Inc., USA).

Experimental data were expressed as column mean, error bars, vertical graphs. The upper and lower whiskers represent the mean ± the standard error of the mean (SEM). The mean is indicated by the line in each graph.

## 3. Results

### 3.1 NK cell receptors are also expressed on CD4^+^ T cells, CD8^+^ T cells, CD19^+^ B cells and CD3^+^CD56^+^ NKT cells present within the fraction of unstimulated peripheral blood mononuclear cells

**Fig 1:**
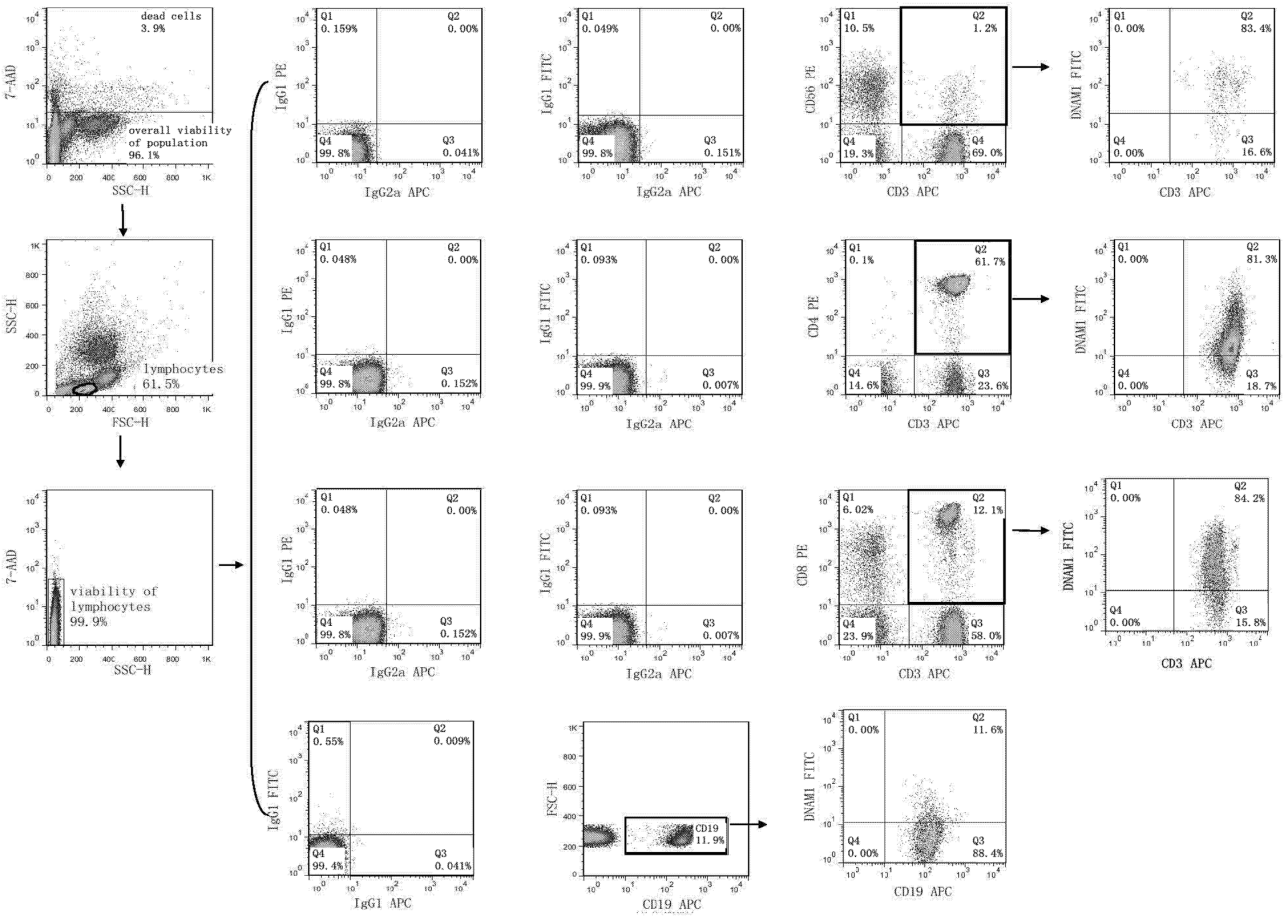
The expression of DNAM-1/CD226 on CD3^+^CD56^+^ NKT cells, CD4^+^ T cells, CD8^+^ T cells and CD19^+^ B cells present within the fraction of unstimulated mononuclear cells immediately after the isolation from peripheral blood of healthy individuals.

**Fig 2:**
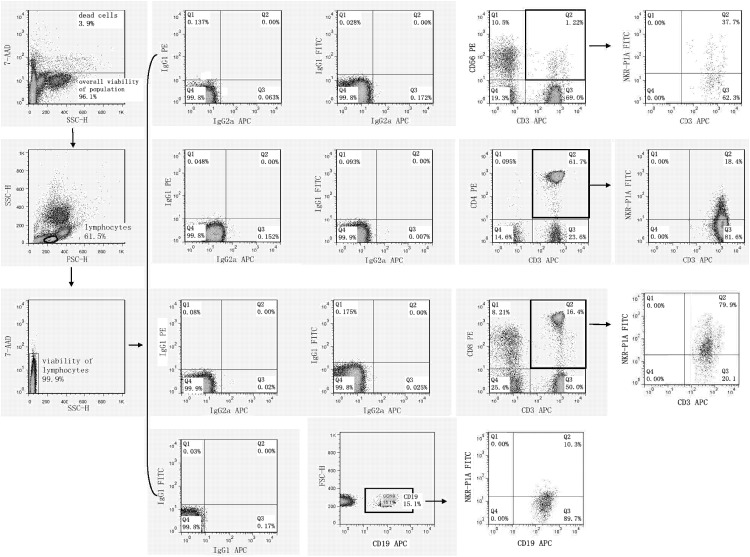
The expression of NKR-P1A/CD161 on CD3^+^CD56^+^ NKT cells, CD4^+^ T cells, CD8^+^ T cells and CD19^+^ B cells present within the fraction of unstimulated mononuclear cells immediately after the isolation from peripheral blood of healthy individuals.

First, we studied the expression of NK receptors on CD4^+^ T cells, CD8^+^ T cells, CD19^+^ B cells and CD3^+^CD56^+^ NKT cells present within the fraction of unstimulated mononuclear cells immediately after the isolation from peripheral blood of healthy individuals. The high expression of DNAM-1 (NK activatory receptor) was detected on CD4^+^ T cells (mean 81.7%), CD8^+^ T cells (mean 84.8%) and NKT cells (mean 81.7%). B cells showed just an intermediate expression of DNAM-1 (mean 12.2%) (Fig 1). While CD8^+^ T cells (mean 78.1%) and NKT cells (mean 40.6%) highly expressed NKR-P1A (NK inhibitory receptor), a moderate expression of NKR-P1A was detected on CD4^+^ T cells (mean 18.1%) and B cells (mean 9.2%) (Fig 2).

The major NK activatory receptor NKG2D was highly expressed mainly on NKT cells (mean 70.4%), nevertheless NKG2D was also found on the cell surface of CD8^+^ T cells (mean 18.3%) and B cells (mean 8.04%). High number of B cells positive for LIR1/ILT-2 (mean 82.6%) was observed, but the NK inhibitory receptor LIR1/ILT-2 showed a certain expression also on CD8^+^ T cells (mean 18.9%). While NKG2A (mean 15.5%), NKG2C (mean 23.0%) and KIR2DL2/L3 (mean 8.4%) were only expressed by NKT cells and NKp80 expression (mean 25.0%) was only demonstrated on CD8^+^ T cells, CD94 and CD16 expression was detected on both CD8^+^ T cells (mean 5.56%, mean 4.54%) and NKT cells (mean 20.5%, mean 9.19%).

Other NK receptors showed no or minimal expression (below 3.5%) on CD4^+^ T cells (CD16, CD94, NKG2D, NKG2C, NKp46, NKp44, NKp30, NKp80, KIR2DL4, LAMP1, NKG2A, KIR2DL2/L3 and LIR1/ILT-2), CD8^+^ T cells (NKG2C, NKp46, NKp44, NKp30, KIR2DL4, LAMP1, NKG2A and KIR2DL2/L3), B cells (CD16, CD94, NKG2C, NKp46, NKp44, NKp30, NKp80, KIR2DL4, LAMP1, NKG2A and KIR2DL2/L3) and NKT cells (NKp30, NKp46, NKp44, NKp80, KIR2DL4, LAMP1 and LIR1/ILT-2) constituting peripheral blood mononuclear cell fraction.

**Fig 3:**
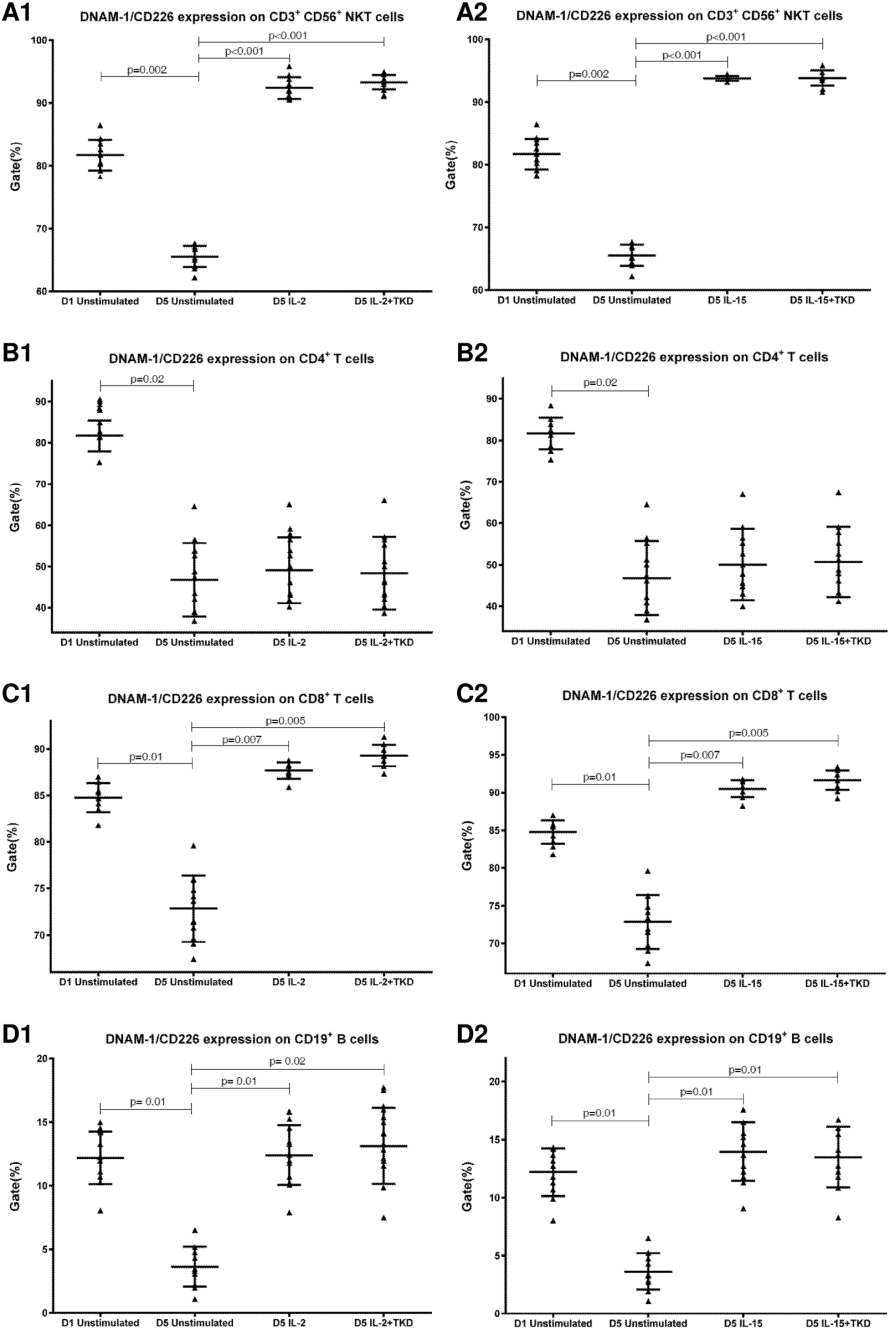
The effect of IL-2, IL-15, IL-2/TKD and IL-15/TKD on the expression of DNAM-1 receptor in peripheral blood mononuclear cells of healthy individuals—the proportion of CD3^+^CD56^+^ NKT cells, CD4^+^ T cells, CD8^+^ T cells and CD19^+^ B cells expressing an appropriate receptor. The expression of DNAM-1 receptor on CD3^+^CD56^+^ NKT cells, CD4^+^ T cells, CD8^+^ T cells and CD19^+^ B cells was examined within the fraction of unstimulated and stimulated mononuclear cells derived from 10 healthy individuals. Data are presented as means ± standard error. In total, the study cohort consisted of 52 Caucasian healthy individuals.

**Fig 4:**
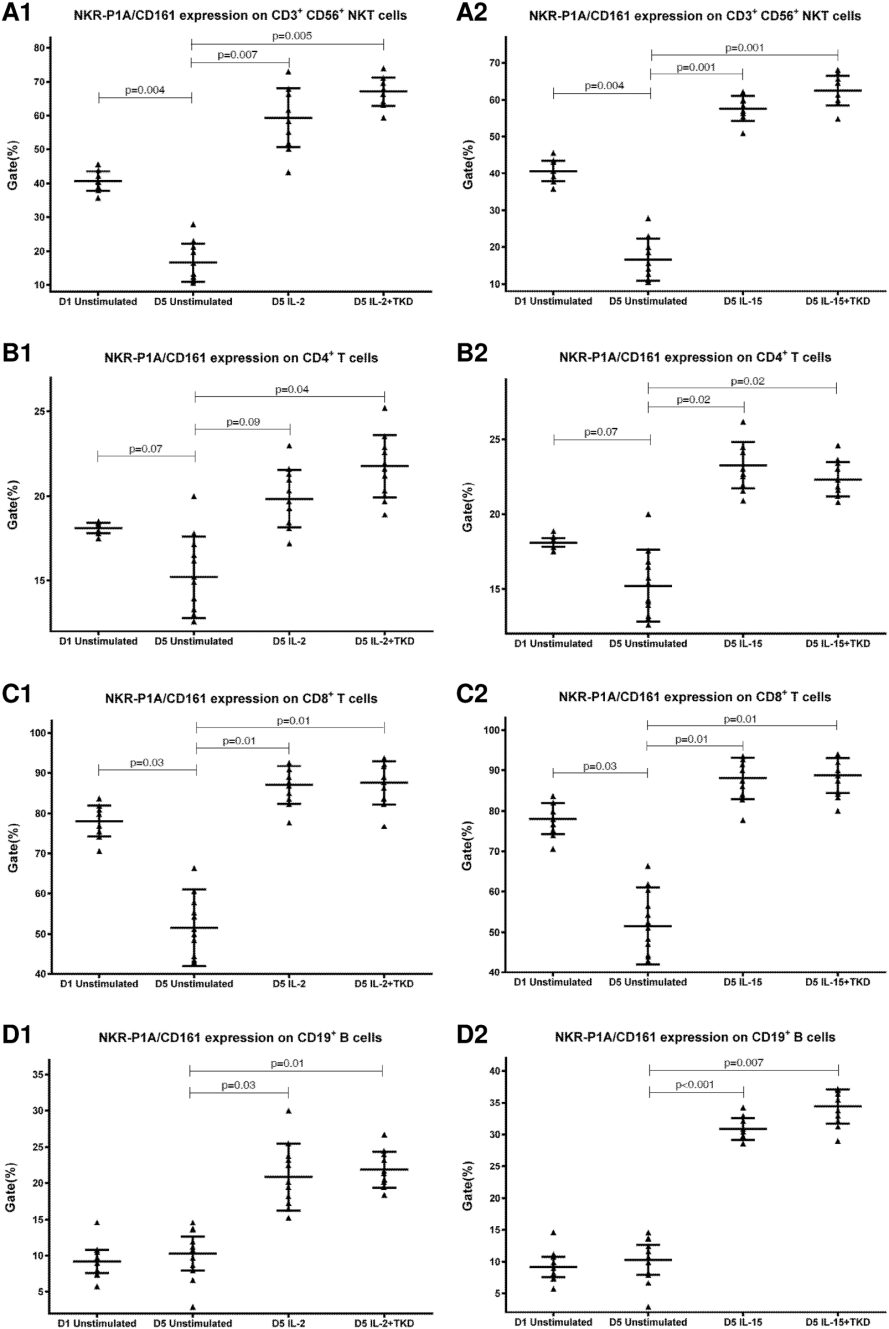
The effect of IL-2, IL-15, IL-2/TKD and IL-15/TKD on the expression of NKR-P1A receptor in peripheral blood mononuclear cells of healthy individuals—the proportion of CD3^+^CD56^+^ NKT cells, CD4^+^ T cells, CD8^+^ T cells and CD19^+^ B cells expressing an appropriate receptor. The expression of NKR-P1A receptor on CD3^+^CD56^+^ NKT cells, CD4^+^ T cells, CD8^+^ T cells and CD19^+^ B cells was examined within the fraction of unstimulated and stimulated mononuclear cells derived from 10 healthy individuals. Data are presented as means ± standard error. In total, the study cohort consisted of 52 Caucasian healthy individuals.

**Fig 5:**
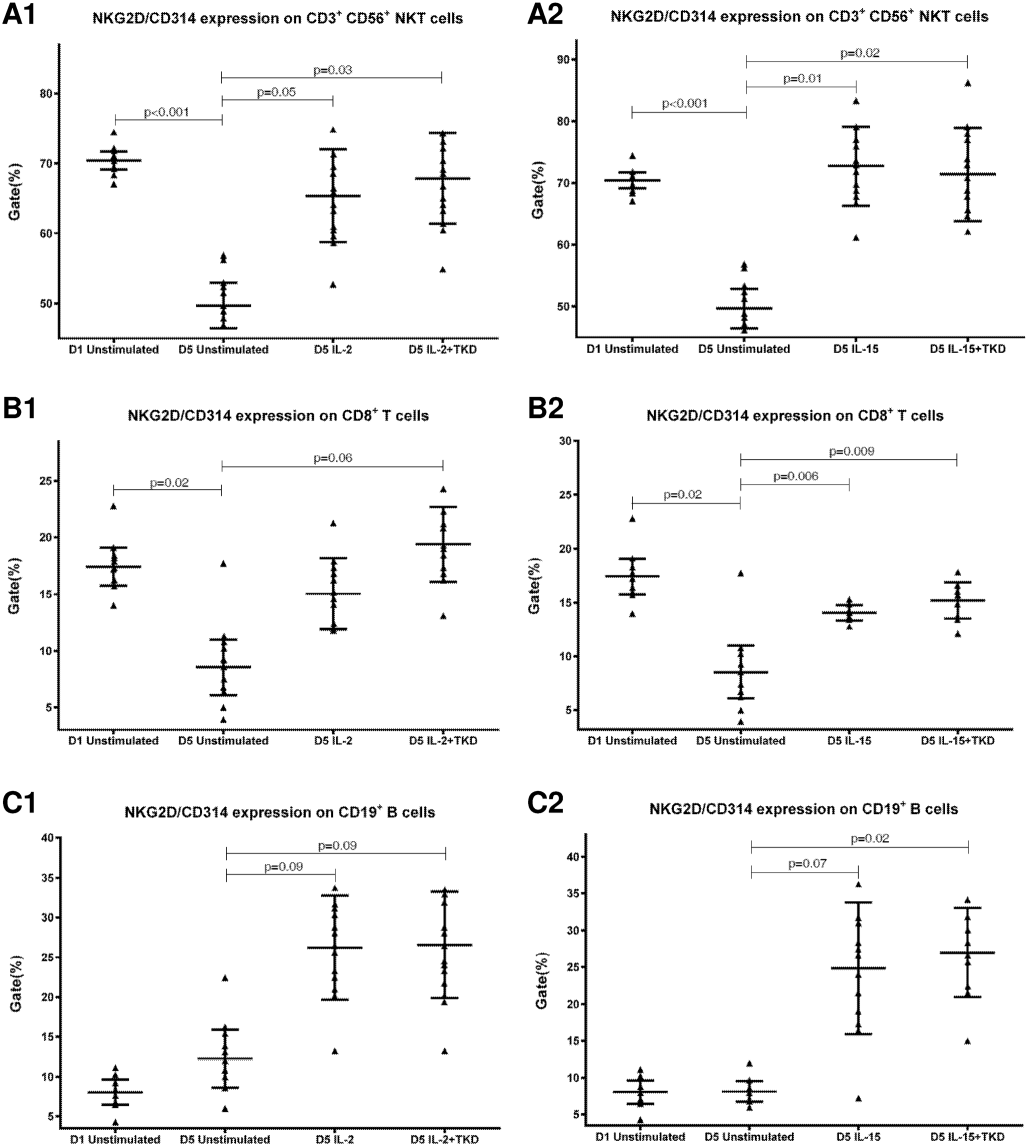
The effect of IL-2, IL-15, IL-2/TKD and IL-15/TKD on the expression of NKG2D receptor in peripheral blood mononuclear cells of healthy individuals—the proportion of CD3^+^CD56^+^ NKT cells, CD8^+^ T cells and CD19^+^ B cells expressing an appropriate receptor. The expression of NKG2D receptor on CD3^+^CD56^+^ NKT cells, CD8^+^ T cells and CD19^+^ B cells was examined within the fraction of unstimulated and stimulated mononuclear cells derived from 10 healthy individuals. Data are presented as means ± standard error. In total, the study cohort consisted of 52 Caucasian healthy individuals.

**Fig 6:**
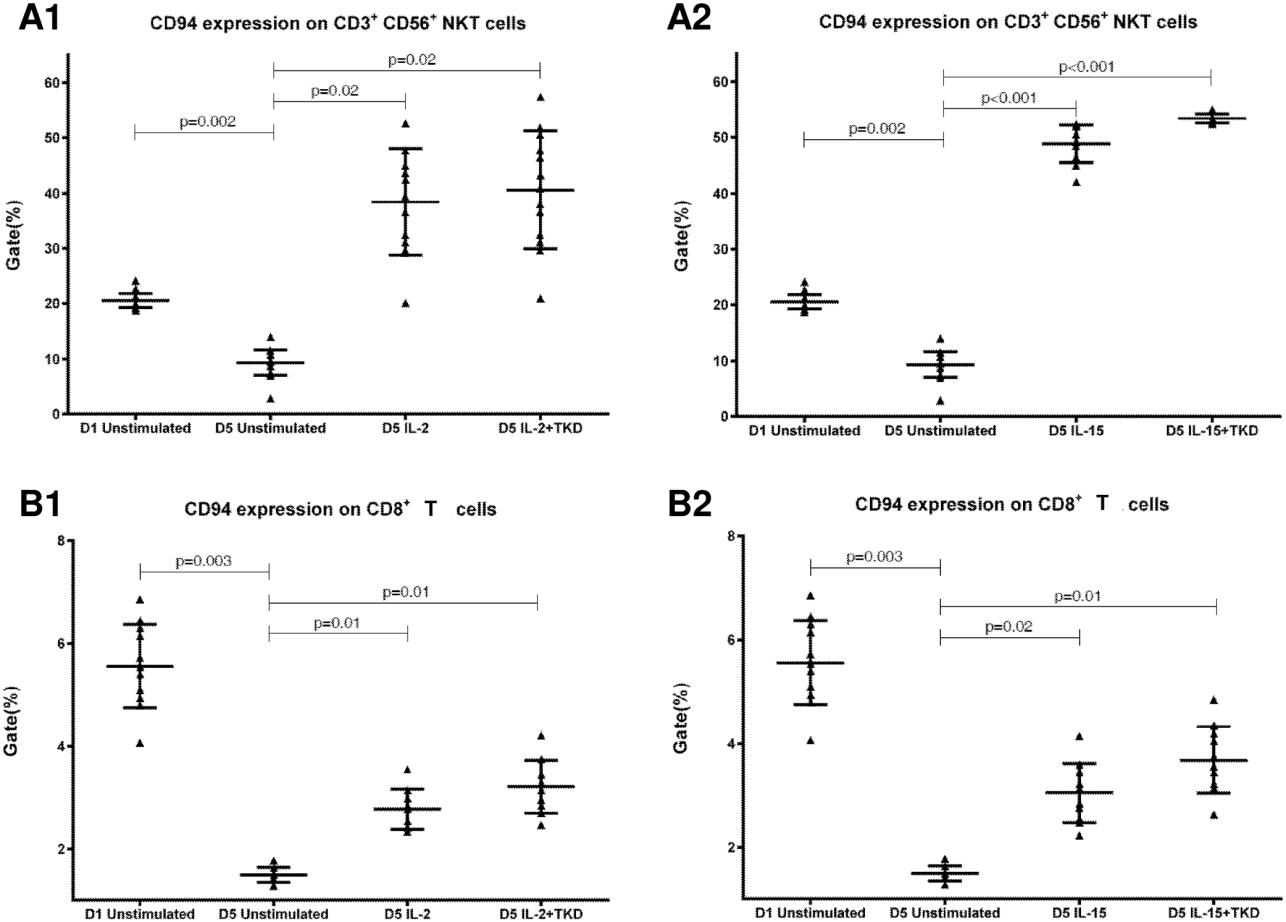
The effect of IL-2, IL-15, IL-2/TKD and IL-15/TKD on the expression of CD94 receptor in peripheral blood mononuclear cells of healthy individuals—the proportion of CD3^+^CD56^+^ NKT cells and CD8^+^ T cells expressing an appropriate receptor. The expression of CD94 receptor on CD3^+^CD56^+^ NKT cells and CD8^+^ T cells was examined within the fraction of unstimulated and stimulated mononuclear cells derived from 10 healthy individuals. Data are presented as means ± standard error. In total, the study cohort consisted of 52 Caucasian healthy individuals.

**Fig 7:**
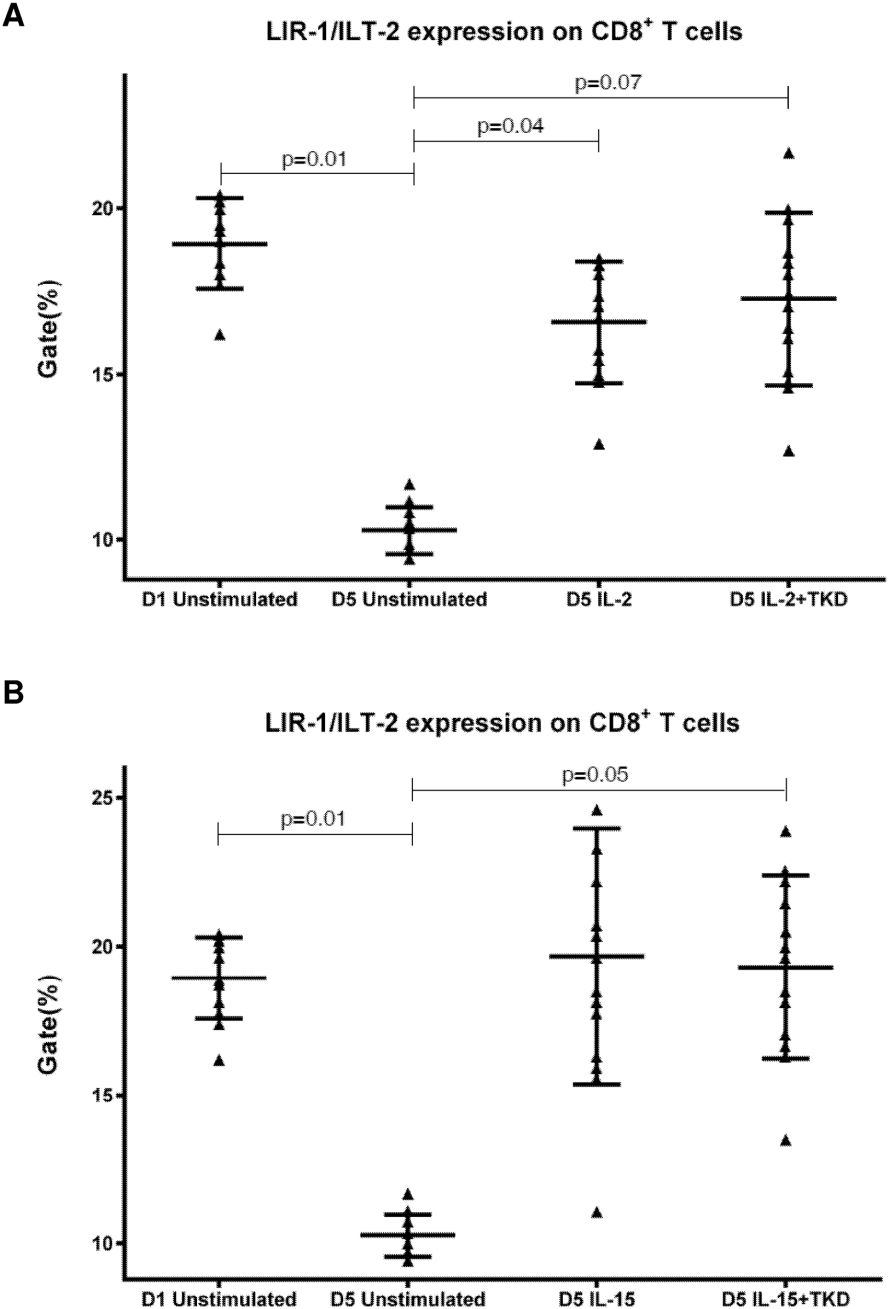
The effect of IL-2, IL-15, IL-2/TKD and IL-15/TKD on the expression of LIR1/ILT-2 receptor in peripheral blood mononuclear cells of healthy individuals—the proportion of CD8^+^ T cells expressing an appropriate receptor. The expression of LIR1/ILT-2 receptor on CD8^+^ T cells was examined within the fraction of unstimulated and stimulated mononuclear cells derived from 10 healthy individuals. Data are presented as means ± standard error. In total, the study cohort consisted of 52 Caucasian healthy individuals.

**Fig 8:**
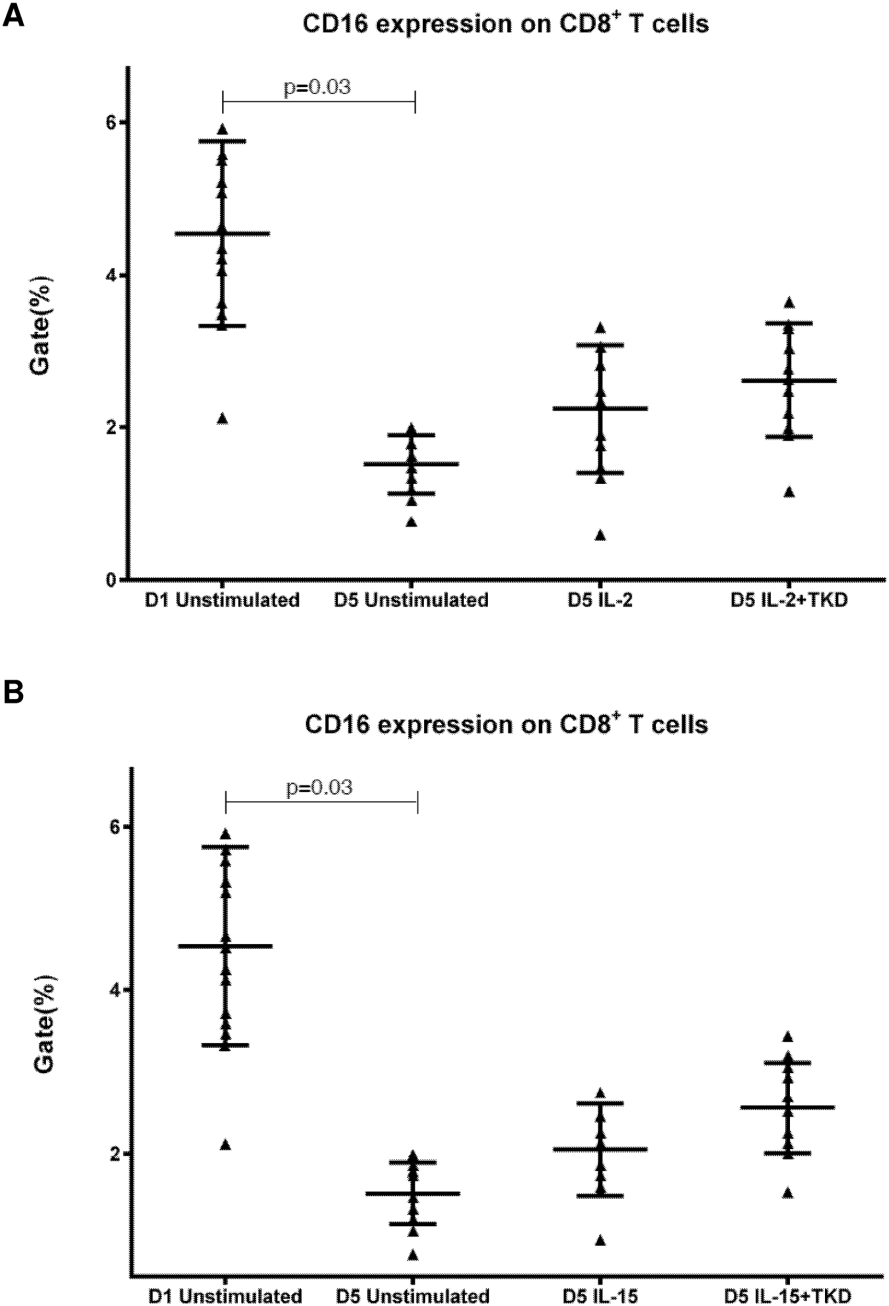
The effect of IL-2, IL-15, IL-2/TKD and IL-15/TKD on the expression of CD16 receptor in peripheral blood mononuclear cells of healthy individuals—the proportion of CD8^+^ T cells expressing an appropriate receptor. The expression of CD16 receptor on CD8^+^ T cells was examined within the fraction of unstimulated and stimulated mononuclear cells derived from 10 healthy individuals. Data are presented as means ± standard error. In total, the study cohort consisted of 52 Caucasian healthy individuals.

**Fig 9:**
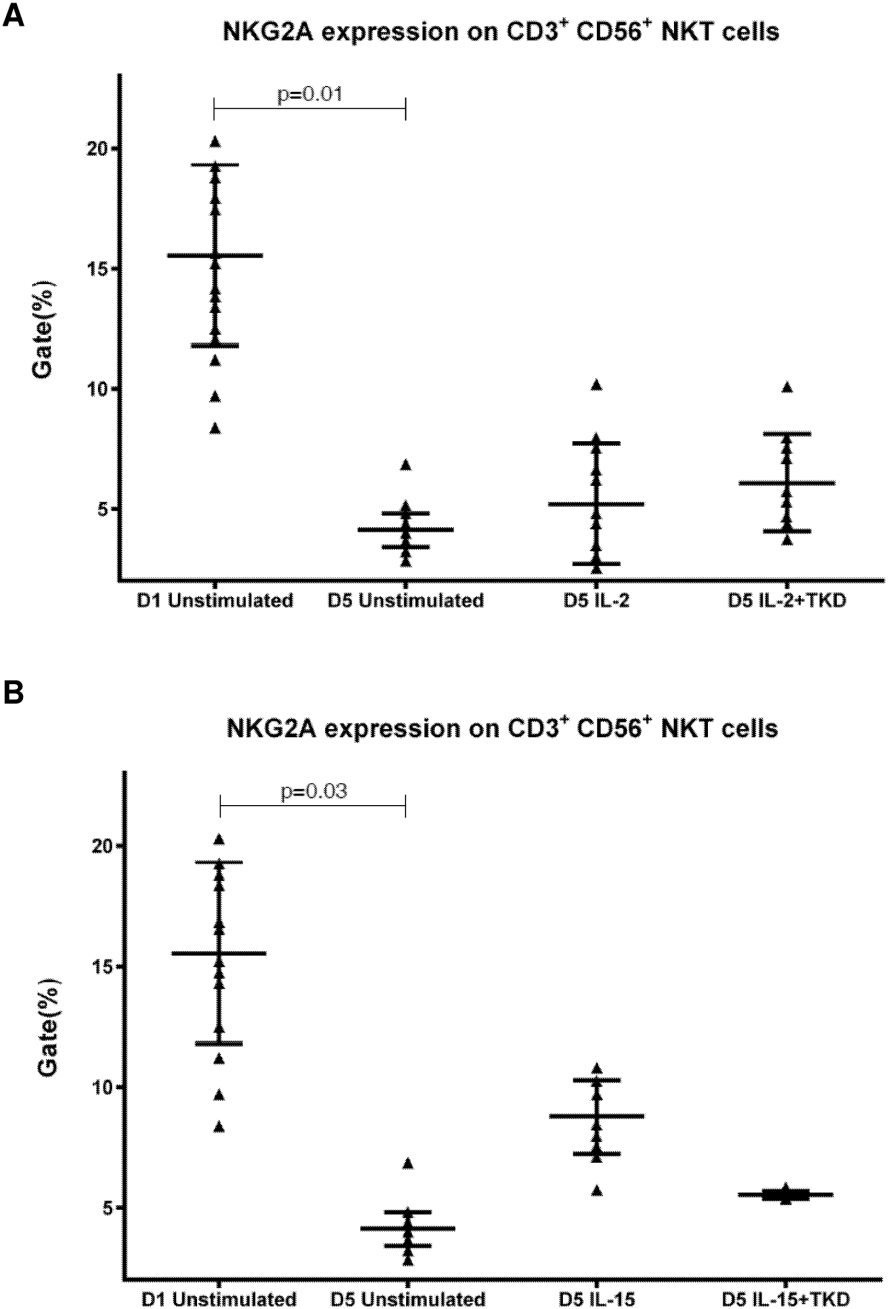
The effect of IL-2, IL-15, IL-2/TKD and IL-15/TKD on the expression of NKG2A receptor in peripheral blood mononuclear cells of healthy individuals—the proportion of CD3^+^CD56^+^ NKT cells expressing an appropriate receptor. The expression of NKG2A receptor on CD3^+^CD56^+^ NKT cells was examined within the fraction of unstimulated and stimulated mononuclear cells derived from 10 healthy individuals. Data are presented as means ± standard error. In total, the study cohort consisted of 52 Caucasian healthy individuals.

**Fig 10:**
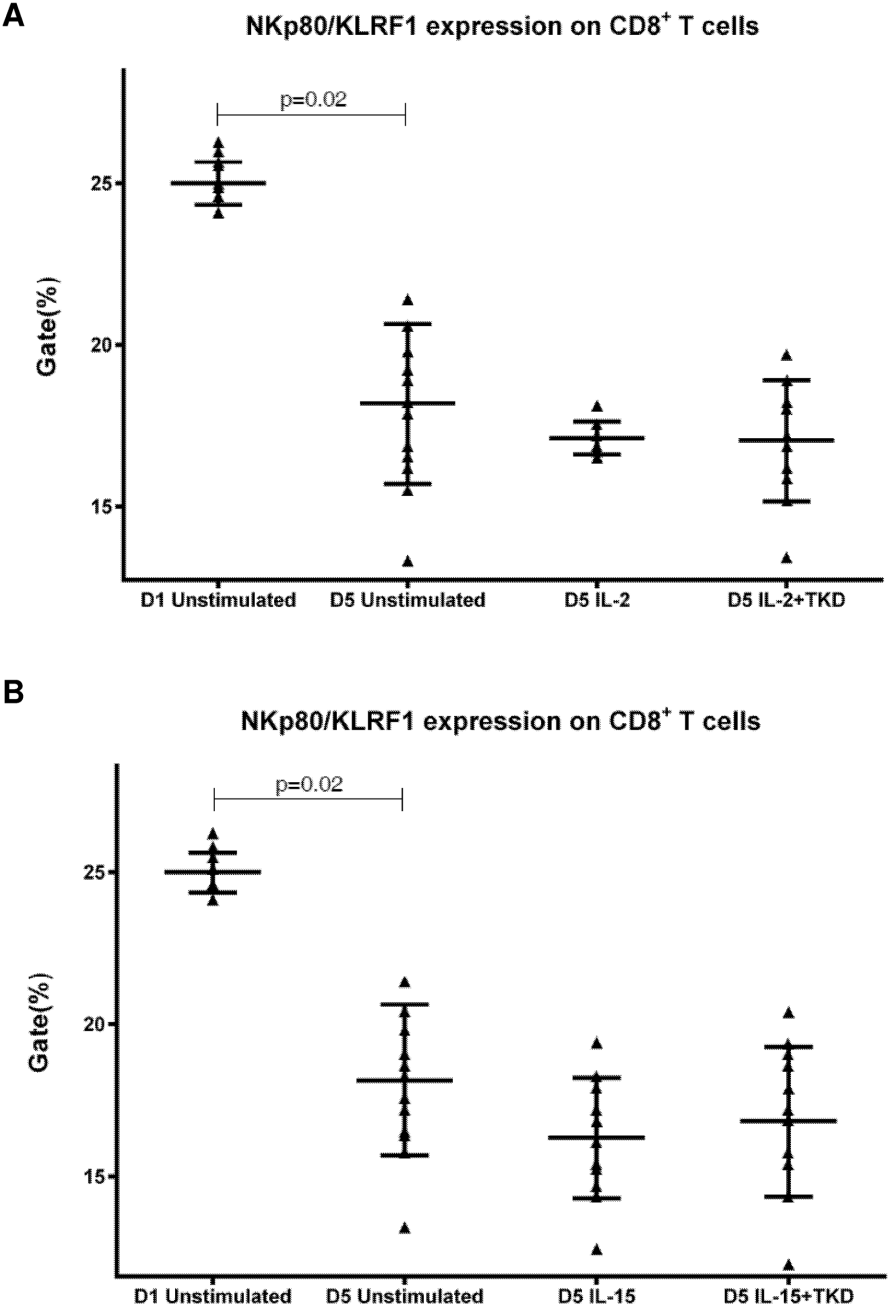
The effect of IL-2, IL-15, IL-2/TKD and IL-15/TKD on the expression of NKp80 receptor in peripheral blood mononuclear cells of healthy individuals—the proportion of CD8^+^ T cells expressing an appropriate receptor. The expression of NKp80 receptor on CD8^+^ T cells was examined within the fraction of unstimulated and stimulated mononuclear cells derived from 10 healthy individuals. Data are presented as means ± standard error. In total, the study cohort consisted of 52 Caucasian healthy individuals.

**Fig 11:**
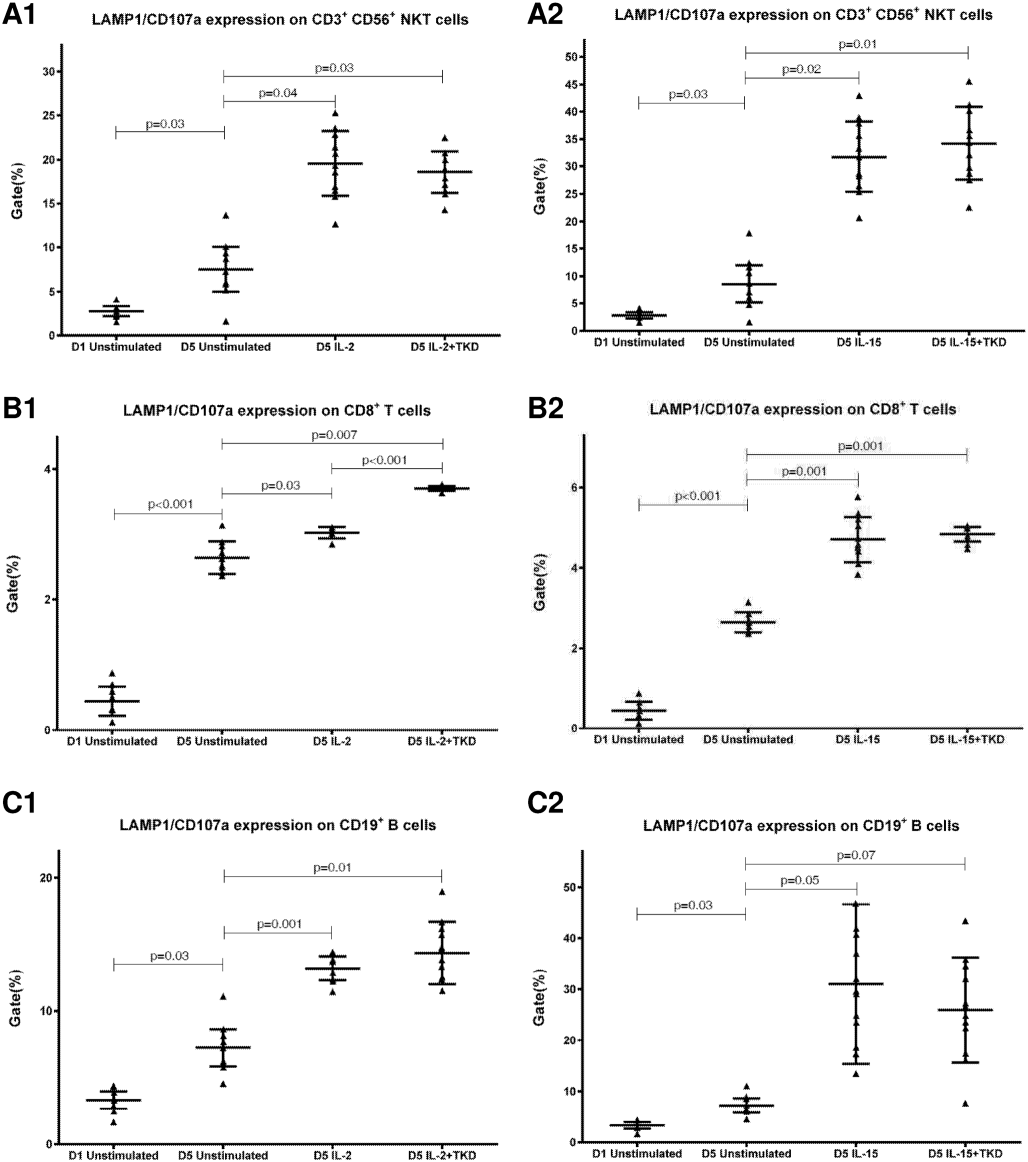
The effect of IL-2, IL-15, IL-2/TKD and IL-15/TKD on the expression of LAMP1 receptor in peripheral blood mononuclear cells of healthy individuals—the proportion of CD3^+^CD56^+^ NKT cells, CD8^+^ T cells and CD19^+^ B cells expressing an appropriate receptor. The expression of LAMP1 receptor on CD3^+^CD56^+^ NKT cells, CD8^+^ T cells and CD19^+^ B cells was examined within the fraction of unstimulated and stimulated mononuclear cells derived from 10 healthy individuals. Data are presented as means ± standard error. In total, the study cohort consisted of 52 Caucasian healthy individuals.

DNAM-1 has been continuously down-regulated in all cell populations (CD4^+^ T cells, CD8^+^ T cells, B cells and NKT cells) with advancing time when peripheral blood mononuclear cells were cultured (flow cytometry analyses performed on day +3 and day +5 of the cell culture) (Fig 3). While NKR-P1A (Fig 4), NKG2D (Fig 5) and CD94 (Fig 6) down-regulation occurred in both CD8^+^ T cells and NKT cells, LIR1/ILT-2 (Fig 7) and CD16 (Fig 8) down-regulation appeared entirely in CD8^+^ T cells. Further, NKG2A (Fig 9) exclusively expressed in NKT cells was also observed to be down-regulated when peripheral blood mononuclear cells had been cultured. Similarly, NKp80 (Fig 10) expressed in CD8^+^ T cells significantly decreased its cell surface expression during the culture of unstimulated peripheral blood mononuclear cells. On the other hand, LAMP1 (Fig 11) showed up-regulation in B cells and NKT cells.

### 3.2 Analysis of cell surface expression of NK cell receptors in relation to interleukines (IL-2 or IL-15) and TKD peptide treatment in CD4^+^ T cells, CD8^+^ T cells, CD19^+^ B cells and CD3^+^CD56^+^ NKT cells present within peripheral blood mononuclear cell fraction

Compared to unstimulated cells, low-dose IL-2 or IL-15 alone and the combination of IL-2/TKD or IL-15/TKD peptide induced after 5-day stimulation a significant upregulation of NKR-P1A on the cell surface of NKT cells, CD4^+^ T cells, CD8^+^ T cells and B cells (Fig 4). Nevertheless, an increase of the proportion of cells expressing DNAM-1 receptor was observed just in case of NKT, CD8^+^ T cells and B cells. CD4^+^ T cells, highly positive for DNAM-1, did not respond to IL-2 or IL-15 alone or in combination with TKD peptide (Fig 3). NKG2D, the major NK cell activatory receptor, was also up-regulated in NKT, CD8^+^ T cells and B cells (Fig 5).

A significant increase of cell number positive for LAMP1 was observed in CD8^+^ T cells, NKT and B cells on day +5 of culture with IL-2 or IL-15 alone or in the mixture with TKD peptide compared to unstimulated cells (Fig 11). Moreover, the difference between IL-2 and IL-2/TKD cell number positive for LAMP1 reached a statistical significance.

CD94 cell surface expression differed before and after the stimulation with IL-2 or IL-15 alone or mixed with TKD peptide in NKT cells and CD8^+^ T cells (Fig 6). Similarly, LIR1/ILT-2 cell surface expression changes were observed in CD8^+^ T cells (Fig 7). The increase of the positive cell numbers for the tested marker was apparent after the treatment with the combination of low-dose IL-2 or IL-15 with or without TKD peptide when compared to unstimulated cells.

Although the increase of the positive cell numbers for the tested marker was apparent after the treatment with the combination of low-dose IL-2 and TKD peptide when compared to IL-2 alone, it does not reach a statistical significance (NKG2D in CD8^+^ T cells) (Fig 5). The effect of IL-15 with TKD peptide on the cell surface expression of appropriate receptors in particular cell subsets was more evident, but once again no statistical difference between IL-15 alone and IL-15/TKD stimulated cells was observed (NKR-P1A and CD94 in NKT cells; NKR-P1A in B cells) (Figs 4 and 6).

### 3.3 The effect of IL-2, IL-15, IL-2/TKD and IL-15/TKD on the expression of DNAM-1, NKR-P1A, NKG2D, CD94, LIR1/ILT-2, CD16, NKG2A, NKp80 and LAMP1 in peripheral blood mononuclear cells of healthy individuals—the amount of receptor expressed by CD3^-^CD56^+^ NK cells, CD3^+^CD56^+^ NKT cells, CD4^+^ T cells, CD8^+^ T cells and CD19^+^ B cells (the median fluorescence intensity)

**Fig 12:**
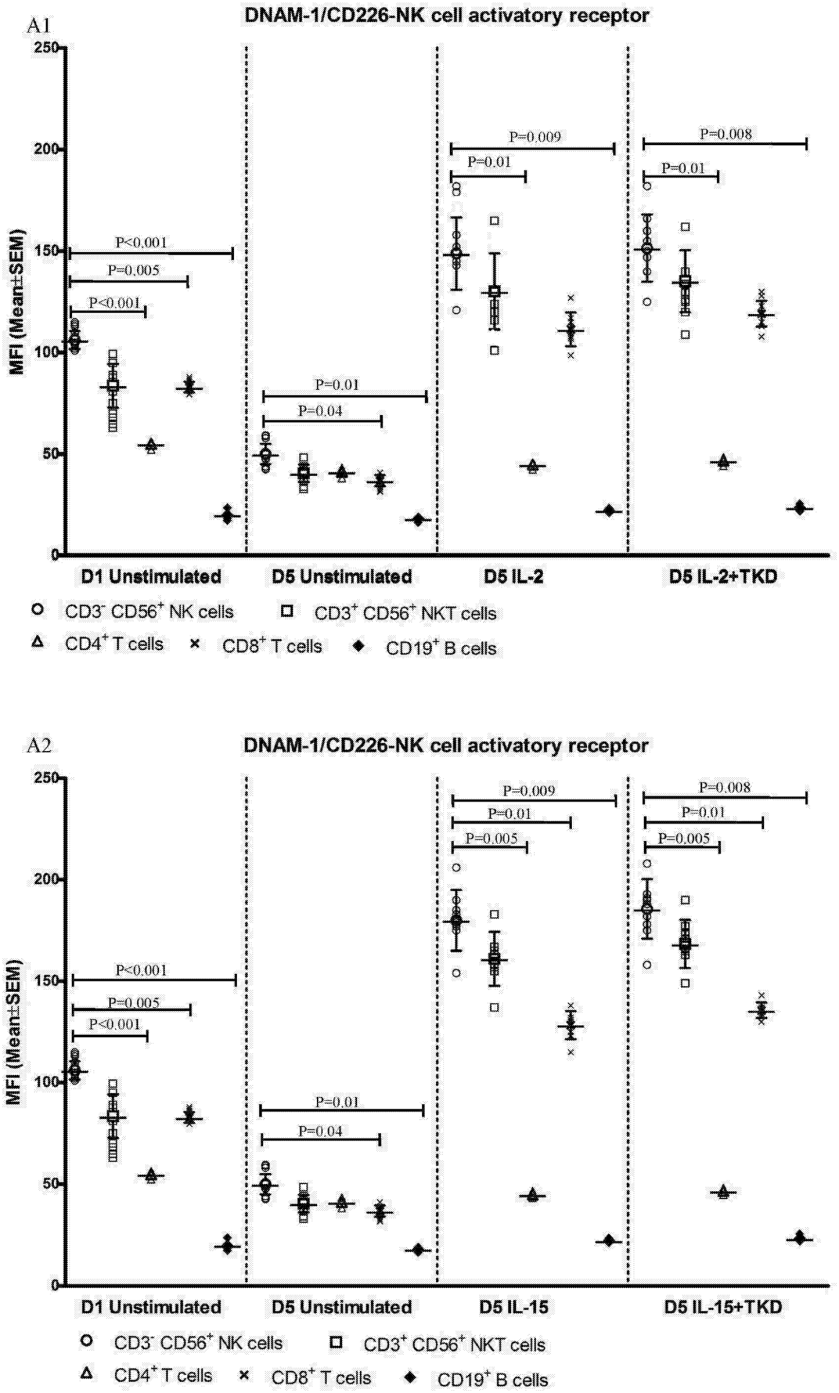
The effect of IL-2, IL-15, IL-2/TKD and IL-15/TKD on the expression of DNAM-1 in peripheral blood mononuclear cells of healthy individuals—the amount of receptor expressed by CD3^-^CD56^+^ NK cells, CD3^+^CD56^+^ NKT cells, CD4^+^ T cells, CD8^+^ T cells and CD19^+^ B cells (the median fluorescence intensity). The amount of DNAM-1 expressed by CD3^-^CD56^+^ NK cells, CD3^+^CD56^+^ NKT cells, CD4^+^ T cells, CD8^+^ T cells and CD19^+^ B cells was examined within the fraction of unstimulated and stimulated mononuclear cells derived from 10 healthy individuals. Data are presented as means ± standard error. In total, the study cohort consisted of 52 Caucasian healthy individuals.

Comparable cell surface density of DNAM-1 was found in IL-2 and IL-2+TKD stimulated CD3^-^CD56^+^ NK cells, CD3^+^CD56^+^ NKT cells and CD8^+^ T cells. Similarly, the MFI values of DNAM-1 were nearly identical in CD3^-^CD56^+^ NK cells and CD3^+^CD56^+^ NKT cells after IL-15 and IL-15+TKD treatment, nevertheless the amount of DNAM-1 receptor expressed by CD8^+^ T cells was significantly lower compared to CD3^-^CD56^+^ NK cells. The lowest DNAM-1 expression was observed in interleukins ± TKD stimulated CD4^+^ T cells and CD19^+^ B cells compared to CD3^-^CD56^+^ NK cells (Fig 12).

**Fig 13:**
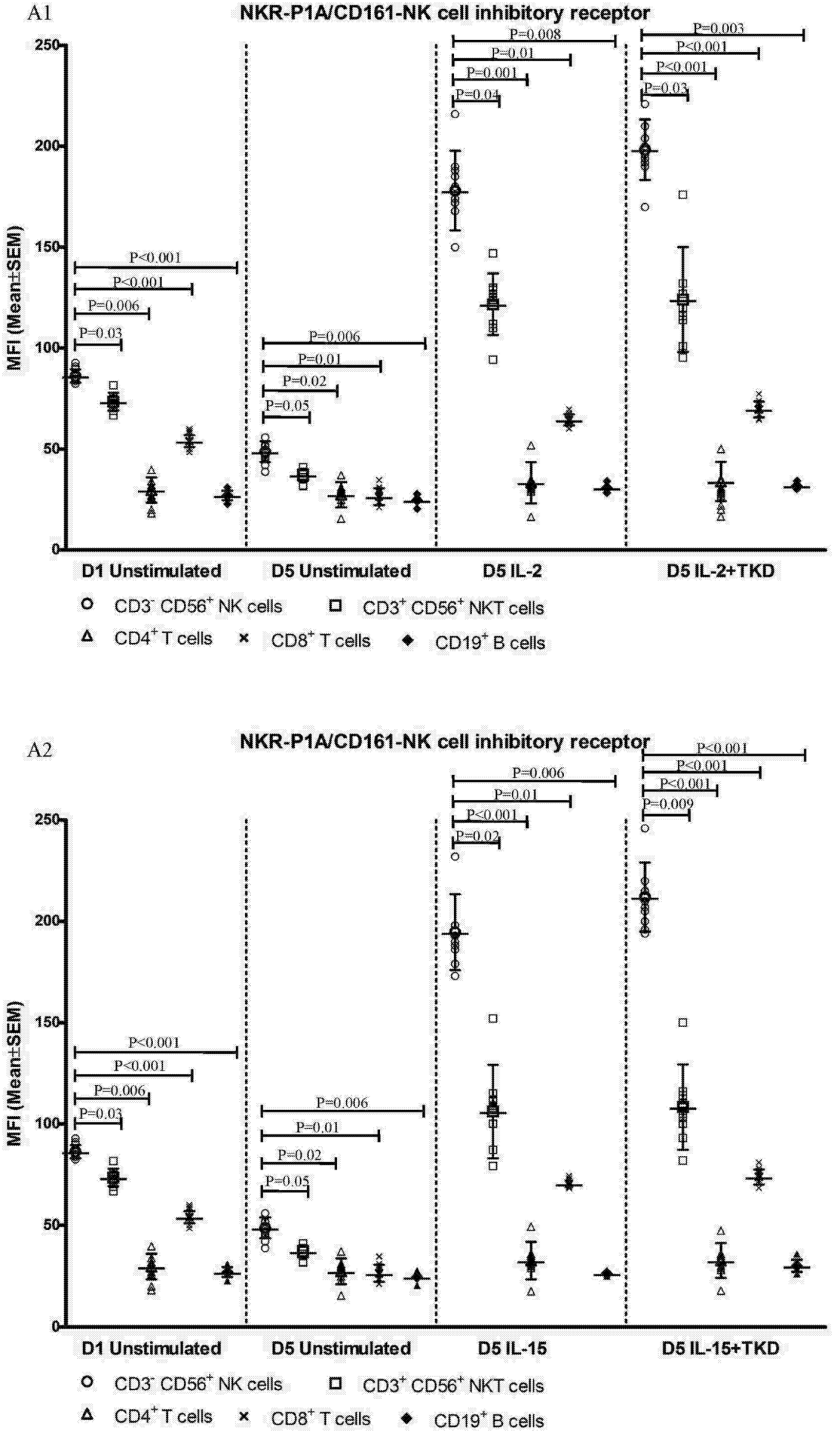
The effect of IL-2, IL-15, IL-2/TKD and IL-15/TKD on the expression of NKR-P1A in peripheral blood mononuclear cells of healthy individuals—the amount of receptor expressed by CD3^-^CD56^+^ NK cells, CD3^+^CD56^+^ NKT cells, CD4^+^ T cells, CD8^+^ T cells and CD19^+^ B cells (the median fluorescence intensity). The amount of NKR-P1A expressed by CD3^-^CD56^+^ NK cells, CD3^+^CD56^+^ NKT cells, CD4^+^ T cells, CD8^+^ T cells and CD19^+^ B cells was examined within the fraction of unstimulated and stimulated mononuclear cells derived from 10 healthy individuals. Data are presented as means ± standard error. In total, the study cohort consisted of 52 Caucasian healthy individuals.

**Fig 14:**
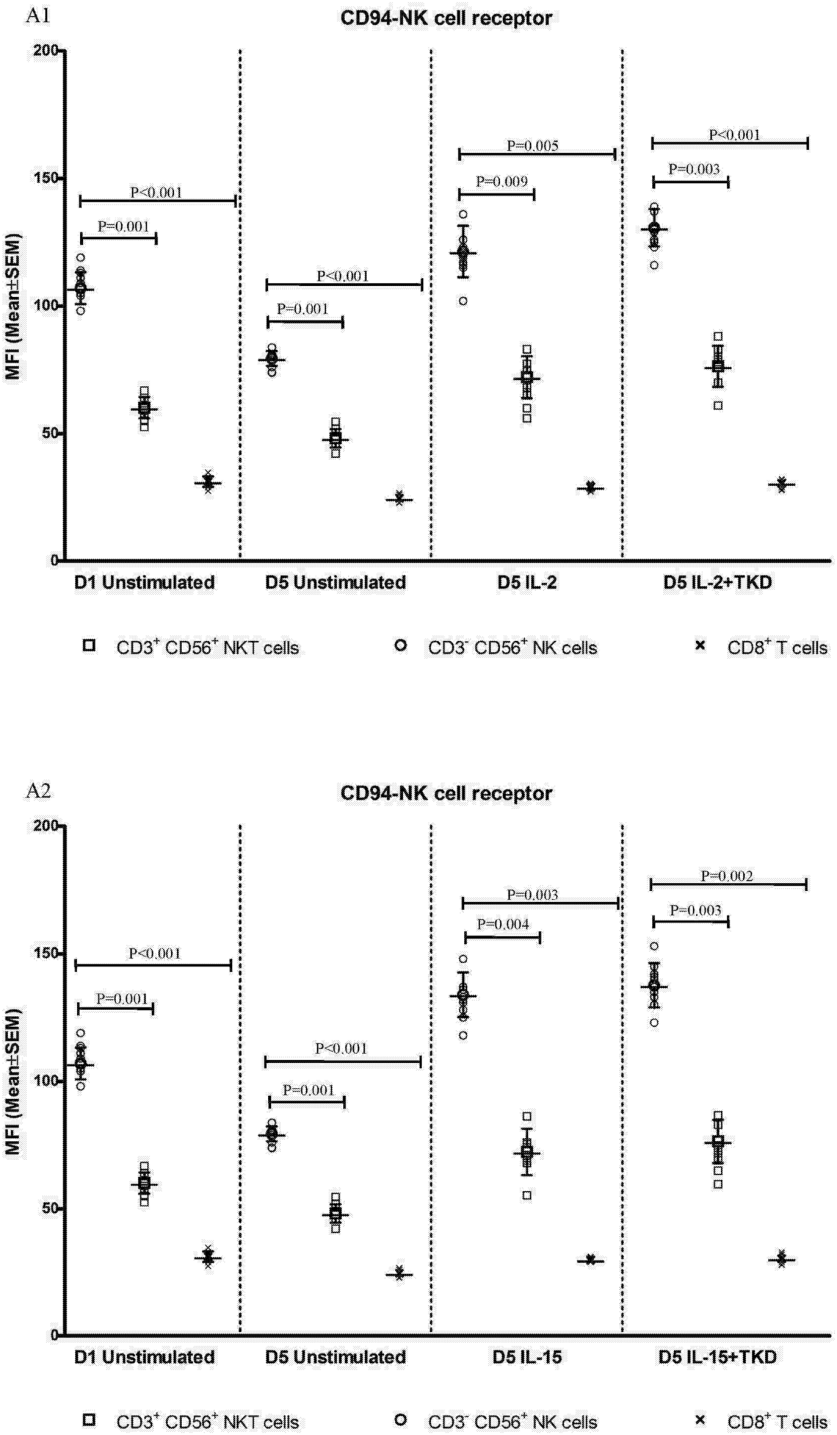
The effect of IL-2, IL-15, IL-2/TKD and IL-15/TKD on the expression of CD94 in peripheral blood mononuclear cells of healthy individuals—the amount of receptor expressed by CD3^-^CD56^+^ NK cells, CD3^+^CD56^+^ NKT cells and CD8^+^ T cells (the median fluorescence intensity). The amount of CD94 expressed by CD3^-^CD56^+^ NK cells, CD3^+^CD56^+^ NKT cells and CD8^+^ T cells was examined within the fraction of unstimulated and stimulated mononuclear cells derived from 10 healthy individuals. Data are presented as means ± standard error. In total, the study cohort consisted of 52 Caucasian healthy individuals.

**Fig 15:**
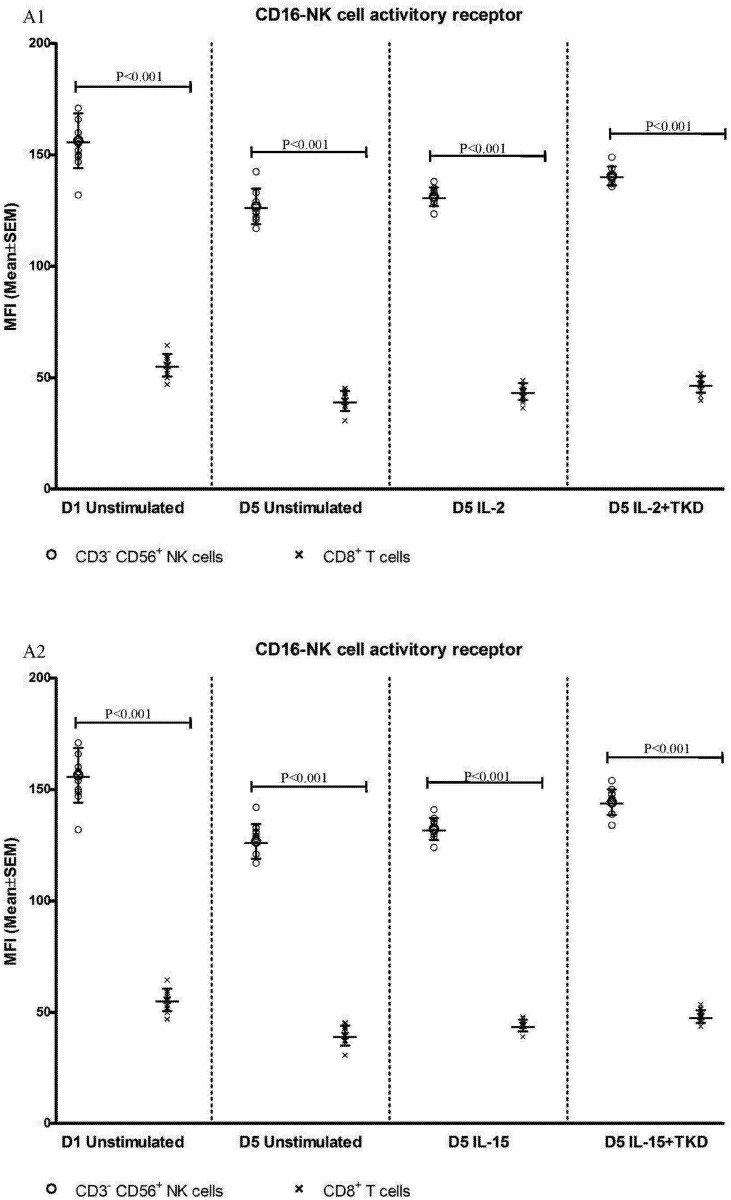
The effect of IL-2, IL-15, IL-2/TKD and IL-15/TKD on the expression of CD16 in peripheral blood mononuclear cells of healthy individuals—the amount of receptor expressed by CD3^-^CD56^+^ NK cells and CD8^+^ T cells (the median fluorescence intensity). The amount of CD16 expressed by CD3^-^CD56^+^ NK cells and CD8^+^ T cells was examined within the fraction of unstimulated and stimulated mononuclear cells derived from 10 healthy individuals. Data are presented as means ± standard error. In total, the study cohort consisted of 52 Caucasian healthy individuals.

**Fig 16:**
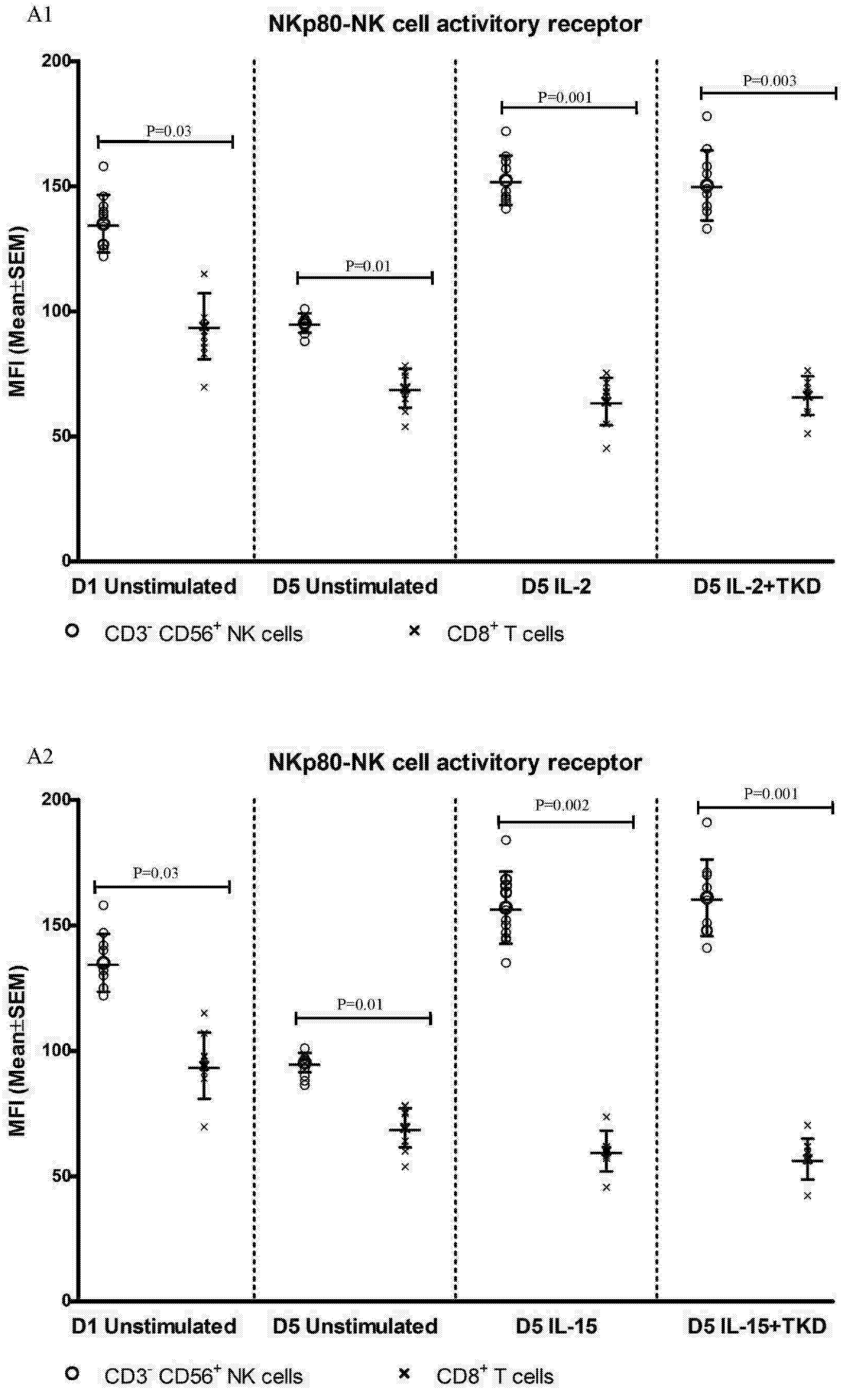
The effect of IL-2, IL-15, IL-2/TKD and IL-15/TKD on the expression of NKp80 in peripheral blood mononuclear cells of healthy individuals—the amount of receptor expressed by CD3^-^CD56^+^ NK cells and CD8^+^ T cells (the median fluorescence intensity). The amount of NKp80 expressed by CD3^-^CD56^+^ NK cells and CD8^+^ T cells was examined within the fraction of unstimulated and stimulated mononuclear cells derived from 10 healthy individuals. Data are presented as means ± standard error. In total, the study cohort consisted of 52 Caucasian healthy individuals.

On the other hand, significantly higher cell surface density of NKR-P1A receptor was observed in CD3^-^CD56^+^ NK cells compared to CD3^+^CD56^+^ NKT cells, CD4^+^ T cells, CD8^+^ T cells and CD19^+^ B cells both before and after stimulation with IL-2/IL-15 alone or in mixture with TKD peptide (Fig 13). Analogous, CD94 receptor demonstrated the highest median fluorescence intensity in both unstimulated and stimulated CD3^-^CD56^+^ NK cells compared to CD3^+^CD56^+^ NKT cells and CD8^+^ T cells (Fig 14). Parallel, the amount of CD16 and NKp80 receptor expressed by unstimulated and stimulated CD3^-^CD56^+^ NK cells was significantly higher than the amount of CD16 receptor expressed by CD8^+^ T cells (Figs 15 and 16).

**Fig 17:**
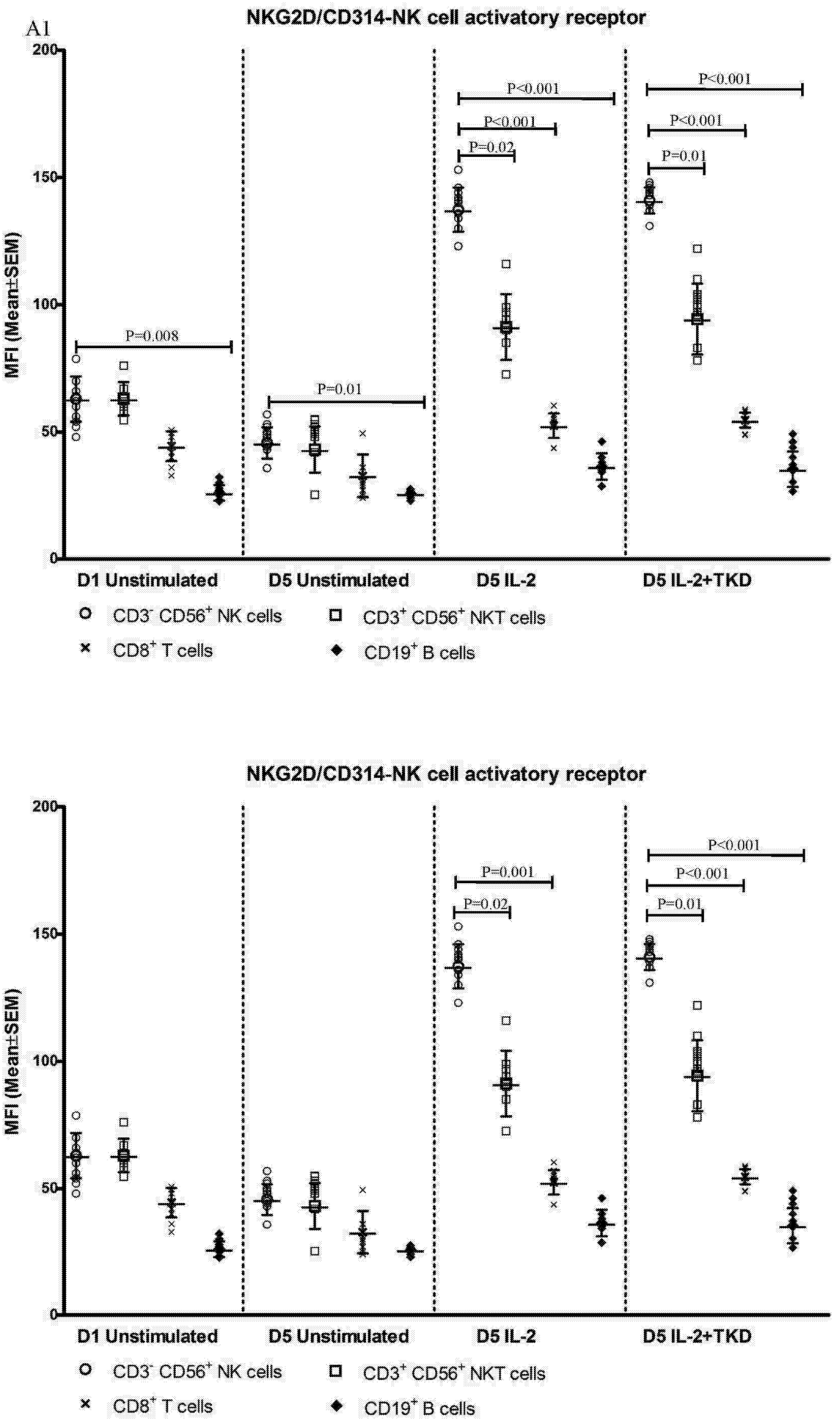
The effect of IL-2, IL-15, IL-2/TKD and IL-15/TKD on the expression of NKG2D in peripheral blood mononuclear cells of healthy individuals—the amount of receptor expressed by CD3^-^CD56^+^ NK cells, CD3^+^CD56^+^ NKT cells, CD8^+^ T cells and CD19^+^ B cells (the median fluorescence intensity). The amount of NKG2D expressed by CD3^-^CD56^+^ NK cells, CD3^+^CD56^+^ NKT cells, CD8^+^ T cells and CD19^+^ B cells was examined within the fraction of unstimulated and stimulated mononuclear cells derived from 10 healthy individuals. Data are presented as means ± standard error. In total, the study cohort consisted of 52 Caucasian healthy individuals.

While the cell surface density of NKG2D receptor was equal in unstimulated CD3^-^CD56^+^ NK, CD3^+^CD56^+^ NKT cells and CD8^+^ T cells, after the treatment with interleukins alone or in combination with TKD peptide the cell surface density of NKG2D receptor was much higher in CD3^-^CD56^+^ NK cells compared to CD3^+^CD56^+^ NKT cells and CD8^+^ T cells (Fig 17). The lowest MFI values of NKG2D were found in both unstimulated and stimulated CD19^+^ B cells (Fig 17).

**Fig 18:**
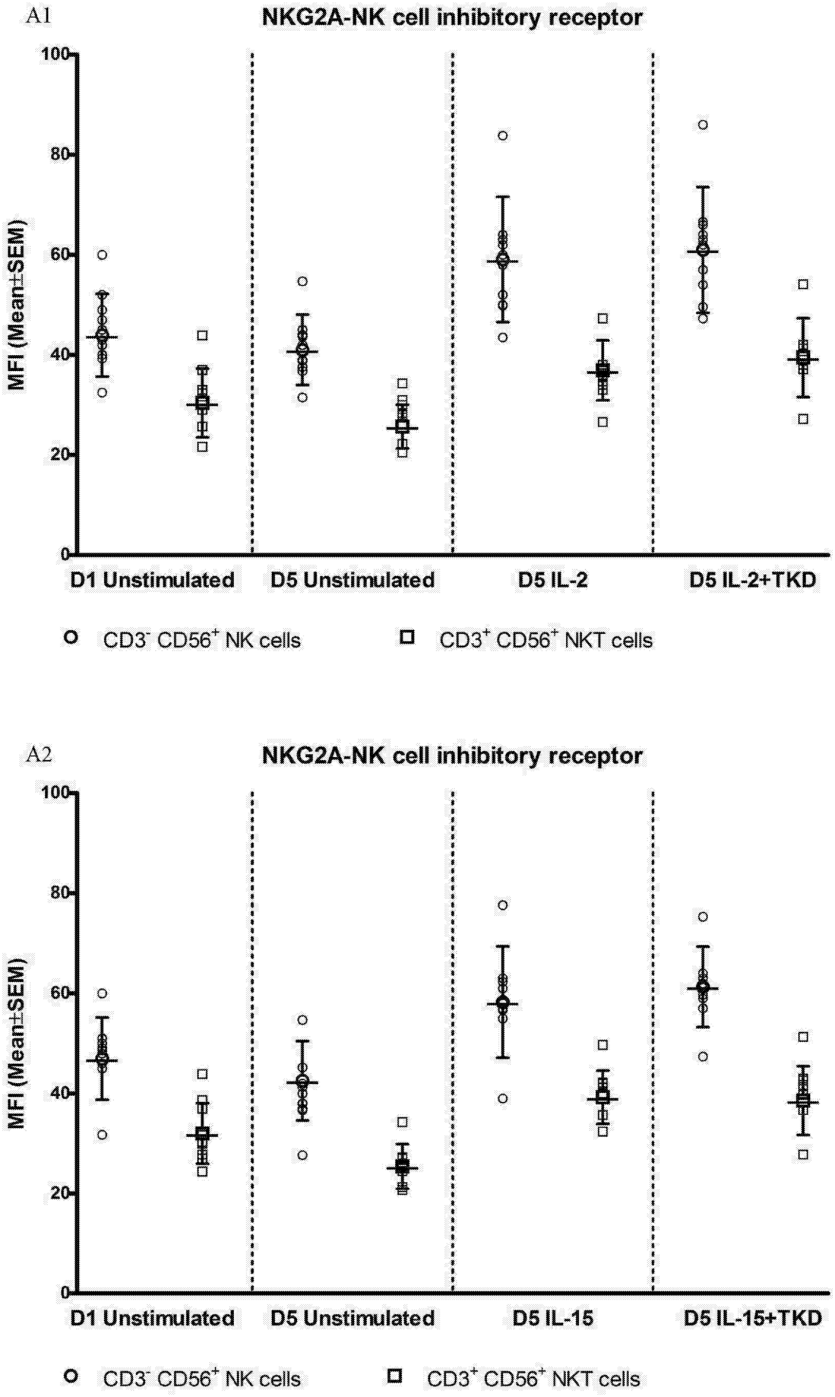
The effect of IL-2, IL-15, IL-2/TKD and IL-15/TKD on the expression of NKG2A in peripheral blood mononuclear cells of healthy individuals—the amount of receptor expressed by CD3^-^CD56^+^ NK cells and CD3^+^CD56^+^ NKT cells (the median fluorescence intensity). The amount of NKG2A expressed by CD3^-^CD56^+^ NK cells and CD3^+^CD56^+^ NKT cells was examined within the fraction of unstimulated and stimulated mononuclear cells derived from 10 healthy individuals. Data are presented as means ± standard error. In total, the study cohort consisted of 52 Caucasian healthy individuals.

**Fig 19:**
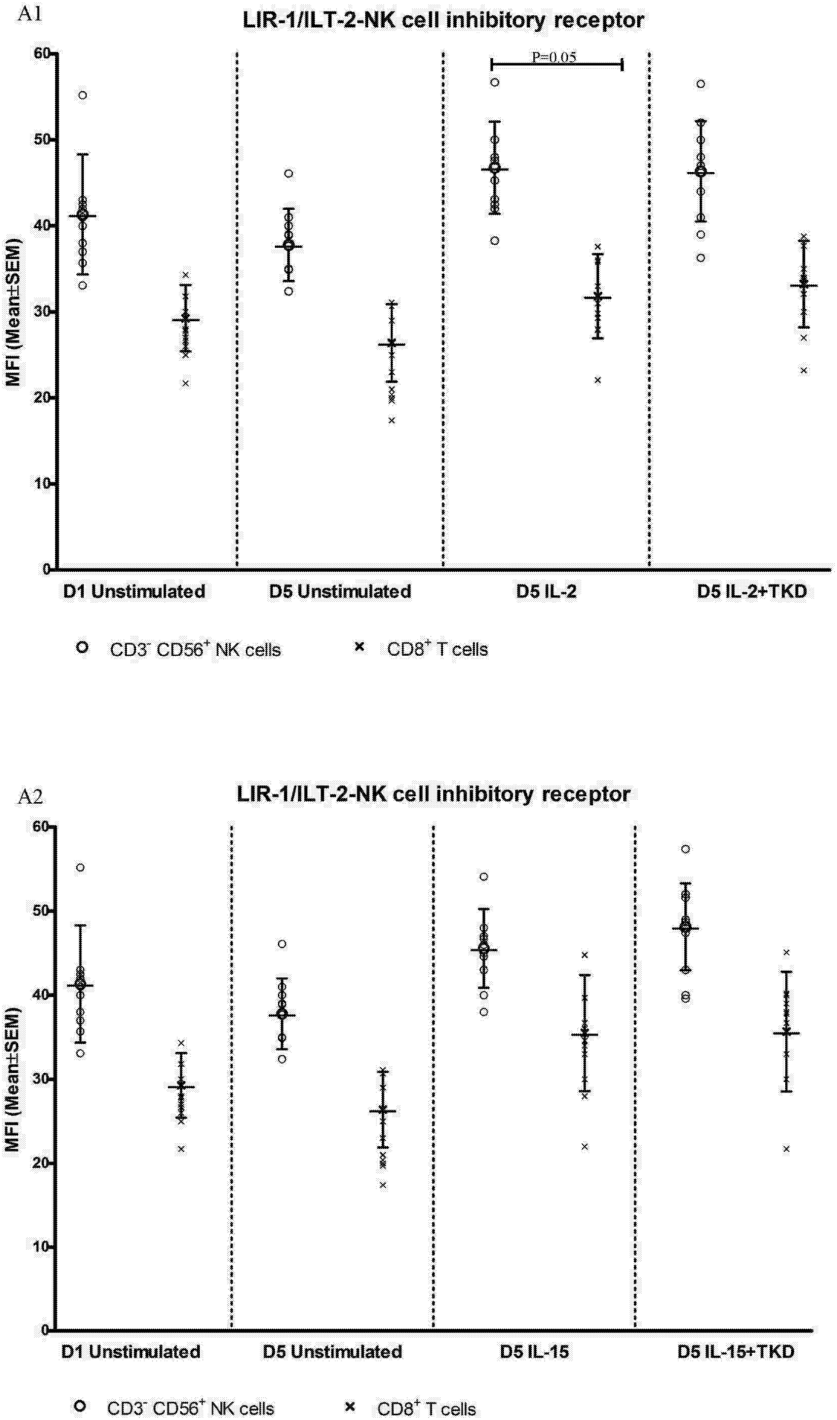
The effect of IL-2, IL-15, IL-2/TKD and IL-15/TKD on the expression of LIR1/ILT-2 in peripheral blood mononuclear cells of healthy individuals—the amount of receptor expressed by CD3^-^CD56^+^ NK cells and CD8^+^ T cells (the median fluorescence intensity). The amount of LIR1/ILT-2 expressed by CD3^-^CD56^+^ NK cells and CD8^+^ T cells was examined within the fraction of unstimulated and stimulated mononuclear cells derived from 10 healthy individuals. Data are presented as means ± standard error. In total, the study cohort consisted of 52 Caucasian healthy individuals.

Interestingly, comparable values of the cell surface density of NKG2A were observed for CD3^-^CD56^+^ NK cells and CD3^+^CD56^+^ NKT cells, when unstimulated or stimulated cells in equal setting were mutually compared (Fig 18). Similarly, the median fluorescence intensity for LIR-1/ILT-2 receptor reached comparable values in CD3^-^CD56^+^ NK cells and CD8^+^ T cells (Fig 19).

**Fig 20:**
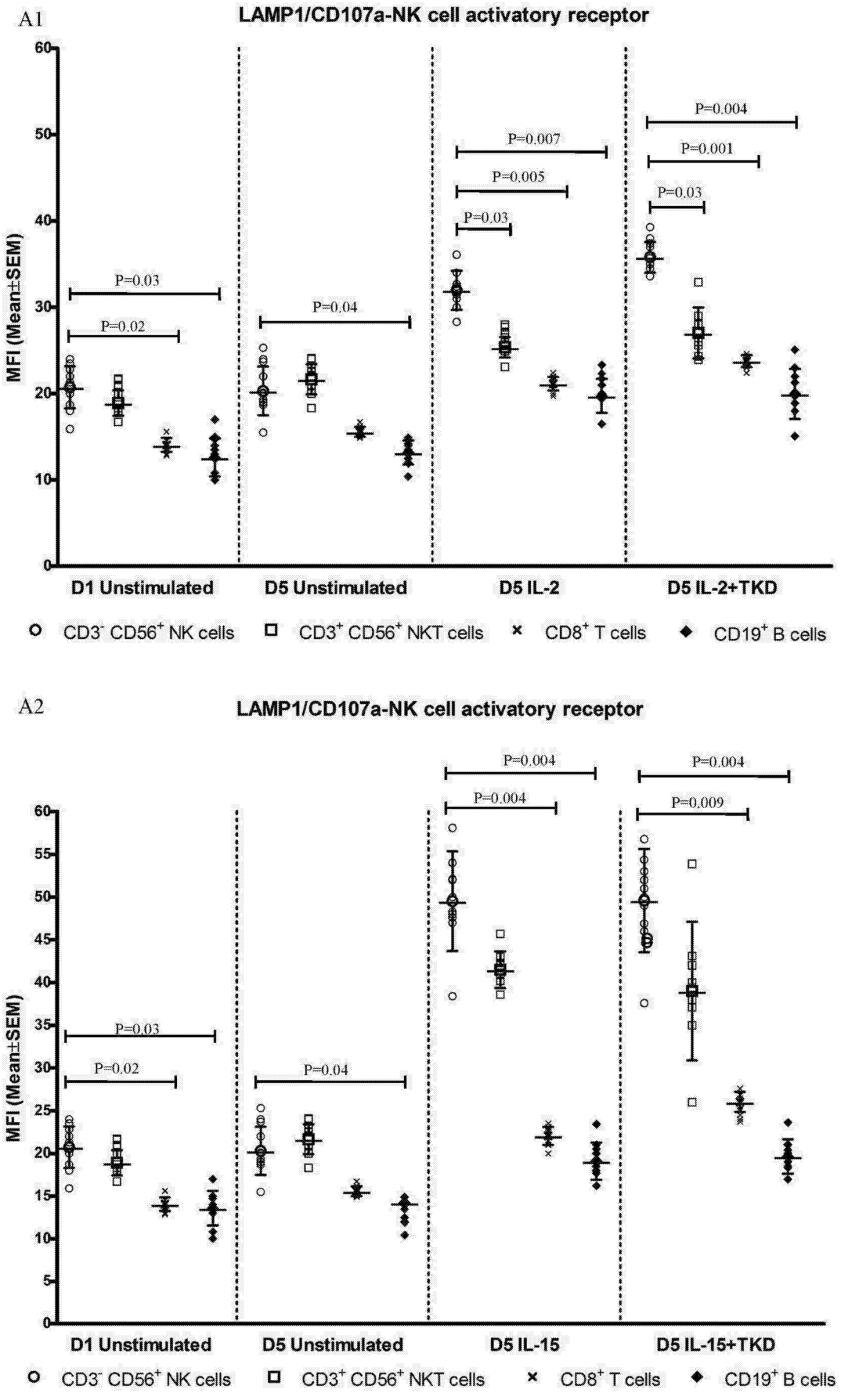
The effect of IL-2, IL-15, IL-2/TKD and IL-15/TKD on the expression of LAMP1 in peripheral blood mononuclear cells of healthy individuals—the amount of receptor expressed by CD3^-^CD56^+^ NK cells, CD3^+^CD56^+^ NKT cells, CD8^+^ T cells and CD19^+^ B cells (the median fluorescence intensity). The amount of LAMP1 expressed by CD3^-^CD56^+^ NK cells, CD3^+^CD56^+^ NKT cells, CD8^+^ T cells and CD19^+^ B cells was examined within the fraction of unstimulated and stimulated mononuclear cells derived from 10 healthy individuals. Data are presented as means ± standard error. In total, the study cohort consisted of 52 Caucasian healthy individuals.

The highest cell surface expression of LAMP1 was identified in IL-2 or IL-15 and IL-2 or IL-15 + TKD stimulated CD3^-^CD56^+^ NK cells compared to CD3^+^CD56^+^ NKT cells, CD8^+^ T cells and CD19^+^ B cells, however the difference between IL-15 ± TKD stimulated CD3^-^CD56^+^ NK cells and CD3^+^CD56^+^ NKT cells did not reach statistical significance (Fig 20).

## 4. Discussion

Previous findings suggest that membrane Hsp70 could serve as an unique immunotherapeutic target for a broad spectrum of tumor entities [18]. The functionality of human NK cells is regulated by interactions of numerous activatory, coactivatory and inhibitory receptors with specific ligands expressed by target cells [19]. NK cell unresponsiveness is under the dominant control of inhibitory receptors that bind with ubiquitously expressed MHC class I molecules. On the other hand, NK cell activation and cytotoxicity is mediated by a deviation in the balance between inhibitory and activatory receptors in favour of activatory receptor signaling [20]. Previous studies of Multhoff group [1,2,3] identified a membrane-bound Hsp70 on tumor cells as a tumor-specific recognition structure for a perforin-independent, granzyme B-mediated attack by allogeneic and autologous NK cells that have been pre-stimulated with Hsp70-derived 14-mer peptide (TKD) in combination with low dose IL-2 [9]. Later, in phase I clinical trial they have shown in patients with metastatic colon carcinoma and non-small cell lung cancer that the application of ex vivo TKD/IL-2 activated autologous leukapheresis product is safe and maintained the cytolytic activity against autologous tumors [10, 11].

The natural killer receptors are also expressed on other cell types found in human peripheral blood including CD4^+^ and CD8^+^ T cells, B cells and monocytes. Since the expression of natural killer receptors is coordinately regulated on divergent cell types as the human immune system maturates, the current study assessed the cell surface expression of CD94, NK activatory receptors (CD16, NKG2D, NKG2C, NKp30, NKp44, NKp46, NKp80, KIR2DL4, DNAM-1, and LAMP1) and NK inhibitory receptors (NKG2A, KIR2DL2/L3, LIR1/ILT-2 and NKR-P1A) on adult healthy individual peripheral blood mononuclear cell populations before and after the stimulation with IL-2 or IL-15 in the presence or absence of hsp70 derived 14-mer peptide (TKD) using flow cytometry. This is the first study demonstrating the effect of IL-2/TKD or IL-15/TKD on cell surface expression of NK cell activatory receptors and NK cell inhibitory receptors in peripheral blood mononuclear cell subsets such as CD3^+^CD56^+^ NKT cells, CD4^+^ T cells, CD8^+^ T cells and CD19^+^ B cells. The effect of in vitro stimulation with IL-2 or IL-15 alone or in combination TKD peptide on cell surface expression of CD94, NK activatory receptors and NK inhibitory receptors in NK cells has been previously reported [12].

Both NK cell activatory receptors and NK cell inhibitory receptors were expressed in other cell subpopulations within the fraction of freshly isolated peripheral blood mononuclear cells. NKT cells were positive for 8 out of 15 studied NK cell receptors (CD16, CD94, NKG2D, NKG2C, DNAM-1, KIR2DL2/DL3, NKG2A and NKRP-1A). CD8^+^ T cells showed the positivity of 7/15 receptors (CD16, CD94, NKG2D, DNAM-1, NKp80, NKRP-1A and LIR1/ILT-2) and B lymphocytes the positivity of 4/15 receptors (NKG2D, DNAM-1, NKRP-1A and LIR1/ILT-2). On the other hand, CD4^+^ T cells were positive just for 2 NK cell receptors (DNAM-1 and NKRP-1A).

CD16, a low affinity IgG receptor, has been already reported to be expressed on monocytes, macrophages, neutrophils, mast cells and besides on NK cells, CD8+ T cells, and peripheral blood NKT-like cells [21, 22], which we confirmed in this independent study. Our data are in agreement with the data of Romero et al. [23] who found that CD4+ resting peripheral blood lymphocytes did not express surface CD94, while CD8+ lymphocytes and NKT-like cells may be positive for CD94 [23, 24, 25]. As expected, we confirmed the presence of NKG2D receptor on unstimulated CD8+ T lymphocytes and NKT cells [26] and the absence of NKG2D receptor on unstimulated CD4+ T lymphocytes derived from healthy individuals [27, 28]. However, we observed a certain expression of NKG2D on unstimulated B lymphocytes as well. We were also able to identify NKG2C positivity on unstimulated NKT cells. Similarly, Lin et al. [29] who used α-GalCer stimulation as a method to obtain large numbers of NKT cells from human peripheral blood, showed the expression of NKG2C on purified NKT cells. DNAM-1/CD226 is constitutively expressed by most NK cells, T helper and cytotoxic T cells and a subset of B cells [30, 31]. These data are in compliance with our current findings. Nevertheless, we also observed a high expression of DNAM-1 on unstimulated NKT cells. In conformity with Kang N et al. and Kuylenstierna et al. we found no expression of KIR2DL2/L3 in unstimulated T cells and B cells [32, 33] and low expression in unstimulated NKT cells [34].

NKG2A receptor has been reported to be expressed in invariant NKT cells distributed preferentially in the liver [35, 36, 37] and maturing CD8^+^ T cells [13]. We could observe a moderate NKG2A expression in NKT cells present within the fraction of unstimulated peripheral blood mononuclear cells. NKRP-1A/CD161 is expressed on most unstimulated NK cells, subsets of CD4+ and CD8+ T cells and NKT cells [38, 39, 40] which is in line with our finding. Moreover, a moderate expression of NKRP-1A/CD161 on unstimulated B cells was detected in the course of the current study.

Our data confirmed the results of the study of Kuttruff et al., who reported that unstimulated CD4+ T cells lacked surface NKp80 and detected NKp80 on a subset of CD8+ T cells at varying frequencies [41]. Compliant with the studies of Colonna et al. LIR1/ILT-2 expression was identified on unstimulated B cells and T lymphocytes [42].

Surprisingly, culturing of unstimulated peripheral blood mononuclear cells induced a significant down-regulation of some examined NK cell receptors expressed on NKT cells (CD94, NKG2D, DNAM-1, NKG2A, NKRP-1A), CD8^+^ T cells (CD16, CD94, NKG2D, DNAM-1, NKp80, NKRP-1A, LIR1/ILT-2), CD4^+^ T cells and B lymphocytes (DNAM-1). On the other hand, LAMP1 receptor was upregulated in unstimulated B cells and NKT cells.

Interestingly, CD94 receptor and NK cell activatory and NK cell inhibitory receptors were upregulated after 5 day stimulation with IL-2 or IL-15 alone or in combination with TKD peptide in other cell populations (CD3^+^CD56^+^ NKT cells, CD4^+^ T cells, CD8^+^ T cells and CD19^+^ B cells) constituting peripheral blood mononuclear cell fraction. An increase in a number of cells with the appropriate receptor on their cell surface was demonstrated in 5 out of 15 examined markers in NKT cells (CD94, NKG2D, DNAM-1, LAMP1, NKRP-1A), 6 out of 15 examined markers in CD8^+^ T cells (CD94, NKG2D, DNAM-1, LAMP1, NKRP-1A, LIR1/ILT-2), 4 out of 15 examined markers in B lymphocytes (NKG2D, DNAM-1, LAMP1, NKRP-1A) and 1 out of 15 examined markers in CD4^+^ T cells (NKRP-1A).

IL-2 and IL-15 have similar biological properties in vitro, consistent with their shared receptor signalling components (IL-2/15βγ_c_). However, specificity for IL-15 versus IL-2 is provided by unique private α-chain receptors that allow differential responsiveness depending on the ligand and high-affinity receptor expressed [43].

The IL-2 stimulation of NK cells leads to a significant up-regulation of all NCRs and NKG2D receptors and to an increase in cytokine secretion, which is associated with enhanced cytotoxic activity against leukemic and tumor cells [44, 45, 46]. Therefore, immunotherapeutic trials with unstimulated and either ex vivo or in vivo IL-2-activated donor NK cells have focused on treating patients with high-risk malignancies in non-transplant settings and after haploidentical stem cell transplantation (haplo-SCT) [46]. However, limited data are available on the effect of IL-2 or IL-15 on other cell populations within in vitro, ex vivo or in vivo stimulated peripheral blood mononuclear cells.

Likewise Dunne et al. who observed expansion and activation of human NK-receptor positive T cells within peripheral blood mononuclear cells cultured for 7 days in the presence of IL-2 or IL-15, we found out that IL-2 and IL-15 expanded NKRP-1A/CD161+ CD8^+^ T cells or CD4^+^ T cells and CD94+ CD8^+^ T cells [47]. Moreover, we demonstrated that IL-2 or IL-15 induced higher proportion of NKT and B cells expressing NKRP-1A/CD161+ receptor and higher proportion of NKT cells expressing CD94 receptor.

Our data are consistent with Mingari et al., who observed the start of CD94 expression in T cells on day 4–6 after the addition of IL-2 or IL-15. Nevertheless, the maximum CD94 expression could be observed on day+10 of the culture [48].

IL-15 and IL-2 up-regulate the expression and function of NKG2D [43, 49, 50]. Probably, as a consequence of this action we were able to observe up-regulation of NKG2D in NKT, CD8^+^ T cells and B cells. IL-15 functions as a key regulator of effector CTL activation and expansion by arming the NKG2D costimulatory pathway under inflammatory conditions [43, 49, 50, 51].

Similarly, cytokine-induced killer cells, CD3+CD56+ terminally differentiated CD8 T cells, generated in vitro by stimulation of peripheral blood mononuclear cells or T-cell subsets with interferon-gamma, anti-CD3 and interleukin-2 have acquired NKG2D, but lack expression of most NK-specific activating (NKp30, NKp44, NKp46) and inhibitory (KIR2DL1, KIR2DL2, KIR3DL1, NKG2A) receptors [52]. Nevertheless, peripheral blood mononuclear cells expanded with IFN-γ, thymoglobulin and IL-2 have already been able to express additional NK activating/inhibitory receptors, such as NKp46, NKG2A, KIR2DL1 and KIR2DL2/DL3 [53].

Our data acquired in the cohort of healthy individuals may be also partially supported by the study of Payne et al. who demonstrated that ex vivo stimulation of peripheral blood mononuclear cells of breast cancer patients with bryostatin 1 and ionomycin combined with IL-2, IL-7, and IL-15 could expand and activate CD25+ NKT and NK cells as well as T memory cells that displayed enhanced reactivity against HER-2/neu positive breast cancer in the presence of myeloid-derived suppressor cells. Ex vivo blockade experiments suggested that the NKG2D pathway might play an important role in overcoming MDSC suppression [54].

LAMP-1/CD107a is a marker for degranulation of NK and activated CD8+ T cells [55]. It was developed as a method to quantitatively assess cytotoxicity of the NK cells and CTL (after overnight activation of PBMCs with IL-2) by measuring the expression of CD107α on the cell membrane, which appeared to be an effective and rapid screening test for cytotoxic defects-related diseases such as familial hemophagocytic lymphopro-liferative syndrome and other hemophagocytic lymphohistiocytosis secondary to primary immunodeficiency [56]. In our study, we observed an increased expression of LAMP-1/CD107a after IL-2 or IL-15 stimulation besides CD8^+^ T cells also on NKT and B cells.

While stimulation of peripheral blood mononuclear cells with IL-2/TKD combination led to the increased expression of NKG2D receptor in cytotoxic lymphocytes only compared to IL-2 alone, more potent effect of IL-15/TKD on peripheral blood mononuclear cell subpopulations was evident in comparison with IL-15 alone. The addition of TKD peptide caused the induction of cell surface expression of CD94, LAMP1 and NKRP-1A receptors in NKT cells and induction of cell surface expression of NKG2D, LIR1/ILT-2 and NKRP-1A receptors in B cells. Nevertheless, this increase has not yet reached a statistical significance.

## 5. Conclusion

In conclusion, the study demonstrated for the first time how other immunocompetent cells present within the fraction of peripheral blood mononuclear cells of healthy individuals that express CD94, NK cell activatory receptors and NK cell inhibitory receptors were influenced under in vitro conditions by the treatment with low-dose interleukins themselves or in combination with hsp70 derived (TKD) peptide enhancing NK cell cytotoxicity toward Hsp70 membrane-positive tumor cells.

## Abbreviations

TKD: Hsp70 derived 14-mer peptide
